# Biodegradable Medical Implants: Reshaping Future Medical Practice

**DOI:** 10.1002/advs.202508014

**Published:** 2025-08-09

**Authors:** Bo Xia, Yan Liu, Yangkun Xing, Zhewei Shi, Xiaocheng Pan

**Affiliations:** ^1^ Department of Bioenvironment Jiyang College of Zhejiang A&F University Zhuji 311800 China; ^2^ Zhuji People's Hospital of Zhejiang Province Zhuji 311800 China

**Keywords:** biodegradable implants, biodegradable materials, medical implants, sustainable biomedical engineering

## Abstract

Biodegradable medical implants have fundamentally transformed the field of biomedical engineering by providing sustainable and biocompatible alternatives that obviate the need for secondary surgical removal and facilitate endogenous tissue regeneration. This comprehensive review systematically evaluates recent advancements in biodegradable implants utilized across a spectrum of medical applications, including internal medicine, surgical interventions, and medical devices. The developments discussed herein signify substantial progress in material synthesis, characterization, and processing techniques, effectively addressing critical challenges associated with the integration of biodegradable devices into medical implants. Although biodegradable medical implants promise advanced patient care, widespread clinical translation is hindered by inconsistent degradation rates mismatched with healing timelines, insufficient load‐bearing strength, and potential inflammatory or toxic by‐products. Additionally, most studies exhibit application‐material imbalances, inadequate in vivo validation, and poorly controlled degradation behavior. Future efforts must clarify degradation mechanisms and treat materials as therapeutic agents. By synthesizing recent findings and highlighting critical gaps, this review provides valuable insights to guide innovation in biodegradable implant technologies, ultimately enhancing clinical outcomes and accelerating regenerative medicine's progress. Addressing these challenges is crucial for realizing their full clinical potential.

## Graphical abstract

Biodegradable medical implants are transforming future healthcare by providing sustainable, biocompatible solutions that eliminate secondary removal procedures, enhancing patients’ physical and psychological comfort, and reducing economic burdens. This review synthesizes recent advances in biodegradable implant research, offering researchers a comprehensive overview and guiding innovations in implant technologies to improve clinical outcomes and advance regenerative medicine.

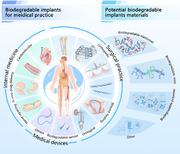

## 1 Introduction

**Scheme 1:**
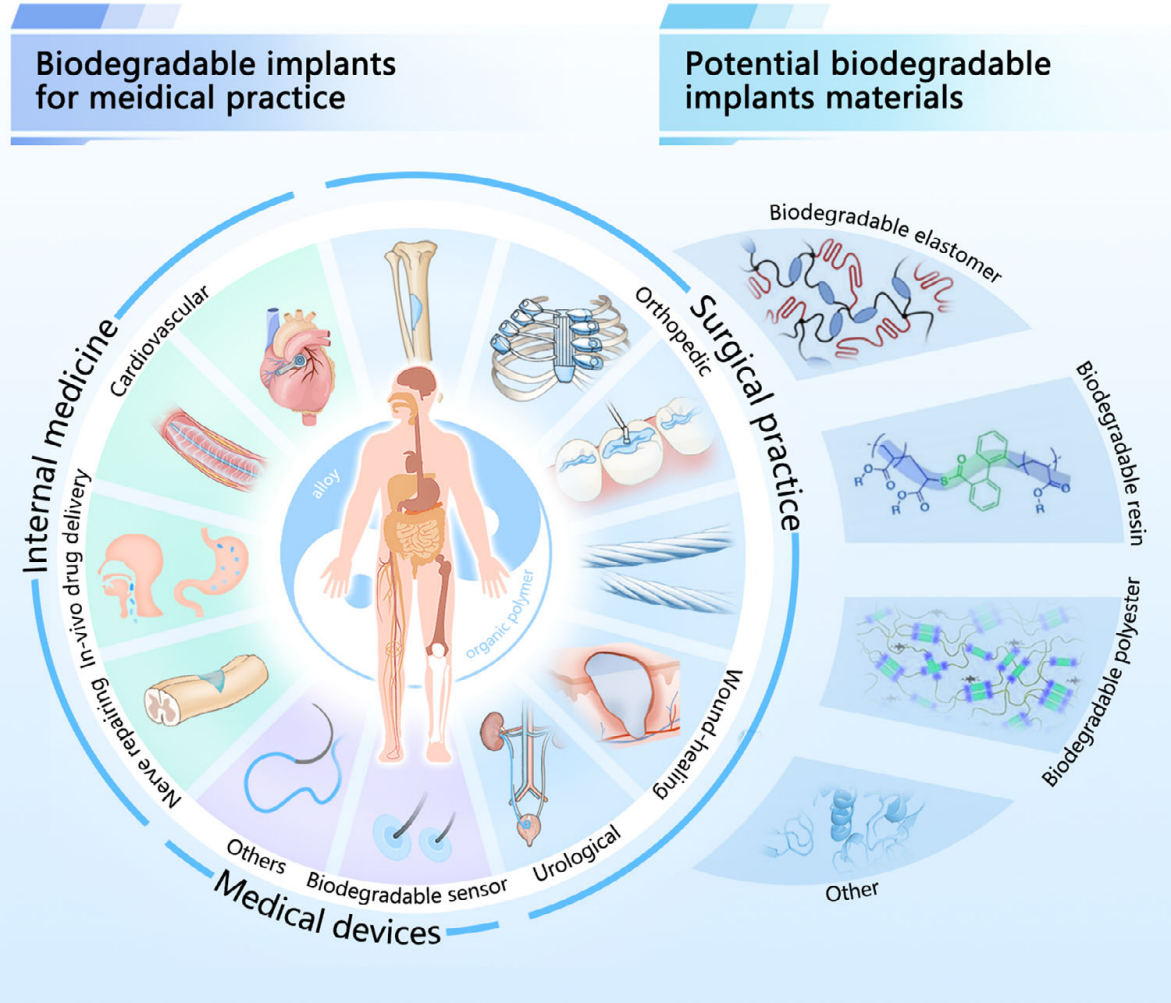
Applications domain of biodegradable implants in medical practice.

Biodegradable medical implants are reshaping future medical practice by offering sustainable, bio‐compatible solutions that eliminate the need for secondary removal procedures, thereby improving patients’ physical and psychological comfort and reducing economic burdens.^[^ ^1^ ^]^ Since their inception, these innovative materials have garnered significant attention from both scientific and clinical communities, paving the way for groundbreaking advancements in biomedical applications. Unlike conventional implants that necessitate removal through secondary surgeries, biodegradable counterparts naturally degrade post‐implementation, thereby minimizing the risk of long‐term complications and additional patient trauma. Moreover, they gradually release bioactive substances during degradation,^[^ ^2^ ^]^ stimulating cellular responses that support soft tissue repair,^[^ ^3^ ^]^ vascular remodeling,^[^ ^4^ ^]^ and bone healing.^[^ ^5^ ^]^ For instance, silk fibroin elastic porous scaffolds have shown remarkable efficacy in enhancing the proliferation of bone marrow stem cells and chondrocytes.^[^ ^6^ ^]^ Another key advantage of biodegradable implants is that mechanical adaptability allows their properties to be engineered to match various tissue requirements, enabling personalized medical solutions. The advent of 3D printing technology further enhances this capability by facilitating the fabrication of customized implants with optimized precision and therapeutic outcomes.^[^ ^7^, ^8^, ^9^, ^10^ ^]^ Over the past decades, biodegradable implants have found widespread use across internal medicine‐such as cardiovascular therapy, nerve repair, and in vivo drug delivery‐as well as in surgical fields including orthopedics, wound healing, and urology, and in various medical devices (**Scheme** **1**). Their material platforms span biodegradable alloys and both natural and synthetic polymers (**Table** **1**). Notably, recent advances in nanotechnology have further enhanced these implants by incorporating nano‐formation‐polymeric nanoparticles, nanogels, and lipid carriers that enable controlled drug release and targeted delivery.^[^ ^11^, ^12^, ^13^, ^14^, ^15^, ^16^ ^]^

**Table 1:** Principal Materials for Biodegradable Implants and Their Application Domains (Grouped by In Vivo Investigation Status).

Material Type	Composition	Application domain	Refs.
Alloys	Mg	Subcutaneous Tissue of the Back	[22]
	ZnO	Cardiovascular Stent Coatings	[23]
	Zn	Femoral Condyle	[24]
	Fe	Proximal Tibia	[25]
	Fe	Proximal Tibia	[5]
	Zn‐1Mg‐3βTCP	Distal Tibia	[26]
	Mg–Zn–Ca	Proximal Tibial Metaphysis	[27]
	Zn‐0.8Li‐0.4Mg	Femoral Condyle	[28]
	Zn–Mg–Cu	Femoral Condyle	[17]
	ZK60 Magnesium	Lateral Condyle of Femur	[29]
	MgOH2 and RS66	Medial Femoral Condyle	[30]
	Zn‐0.5 V, Zn‐0.5Cr, and Zn‐0.5Zr	Femoral Condyle, Abdominal Aorta	[31]
	Iron‐Manganese	Cranium	[32]
	Fe‐Mn‐xCu	Femur	[33]
	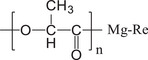	Osteosarcoma Cells	[34]
Natural/modified natural polymers	Val‐Pro‐Gly‐Xaa‐Gly modified silk fibroin	Subcutaneous Dorsal, Trochlear Groove of Femur in Knee Joint	[35]
	gelatin/HA/placental extract	Mastoid Process of Temporal Bone	[36]
	Whey Protein	Subcutaneous Tissue on the Back	[37]
	Modified silk fibroin	Vascular Endothelial Cells	[22]
Natural/modified natural polymers	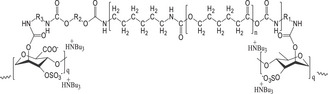	Subcutaneous Dorsal	[38]
	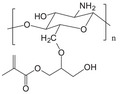	Human Colorectal Adenocarcinoma Cells	[39]
	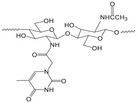	Full‐Thickness Skin on the Dorsum	[40]
	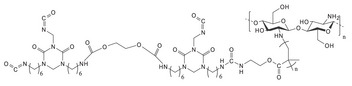	Subcutaneous Tissue	[41]
	Poly (glycerol sebacate) derivatives	Carotid Artery	[42]
	Zein/polyvinylpyrrolidone	Subcutaneous Tissue on the Back	[43]
	Chitosan and poly (lactic‐co‐glycolic Acid)	Sciatic Nerve	[44]
	Polycaprolactone microparticles with molybdenum	Sciatic Nerve	[45]
Synthetic polymers	High‐molecular‐weight hyaluronan with procyanidine and Fe^3+^	Intestinal	[46]
	Decellularized platelet‐rich fibrin‐loaded zinc‐doped magnesium phosphate	Femoral Condyle	[47]
	Nano‐sheet (H‐Si) @ hydroxyapatite (HA)‐coated @ Ti	Right Tibia	[48]
	Poly (3‐sulfopropyl methacrylate potassium salt) @ Cu^2+^	Back Subcutaneous Tissue	[49]
	Liquid Crystal Elastomer: pentaerythritol tetrakis (3‐mercaptopropionate)/1,4‐bis‐[4‐(3‐acryloyloxypropyloxy)benzoyloxy]‐2‐methylbenzen	Dorsal Skin	[50]
	Liquid Crystal Elastomer: 1,4‐bis‐[4‐(6‐acryloyloxyhexyloxy) benzoyloxy]‐2‐methylbenzene/2,2′‐ (ethylenedioxy) diethanethiol/1,3,5‐Triallyl‐1,3,5‐triazine‐2,4,6(1H,3H,5H)‐trione	Bladder Neck	[51]
	Injectable conductive hydrogels (ICHs)	Sciatic nerve	[52]
	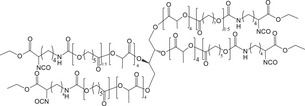	Soft Tissue Defects	[3]
	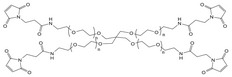	Subcutaneous Tissue	[4]
	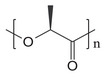	Skull	[53]
Synthetic polymers	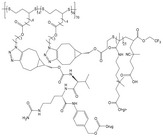	Triple‐Negative Breast Cancer Cells	[19]
	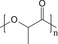	Endovascular	[54]
	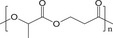	Sciatic Nerve	[55]
	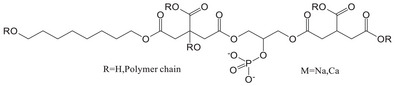	Bilateral Lateral Femoral Condyles	[56]
	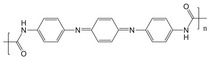	Skull	[57]

Despite these advancements, biodegradable implant materials still face significant challenges. Achieving an optimal balance between degradation rate and mechanical integrity is critical; materials that degrade too quickly may compromise structural support during healing, while those degrading too slowly can impede timely tissue regeneration. This issue is particularly pronounced in orthopedic applications, where implants must sustain bone healing before complete resorption.^[^ ^17^ ^]^ Additionally, biodegradable materials often lag metals in strength and durability, especially in high‐load‐bearing applications like weight‐bearing bones, posing a barrier to their widespread adoption. Economic challenges also persist, such as synthesizing high‐purity biodegradable materials that require complex, resource‐intensive processes, further burdened by stringent sterilization protocols.^[^ ^18^ ^]^ These factors lead to elevated production costs, limiting accessibility and scalability. Environmental concerns related to raw material extraction and processing are significant, particularly in the context of global sustainability goals. Moreover, inconsistent regulations across regions and institutions hinder the uniform adoption of biodegradable implants, creating market uncertainty and complicating clinical implementation.^[^ ^19^ ^]^ Thus, ensuring biocompatibility and safety remains a major hurdle for the medical community.

Based on these advantages and challenges, this review comprehensively examines the applications, preparation methods, and developments of biodegradable materials in the medical field over the past five years, highlighting their significance in modern healthcare. Unlike previous reviews that typically concentrate on specific materials or applications‐for instance, notable reviews on cardiac piezoelectric or biodegradable medical piezoelectric implants^[^ ^20^, ^21^ ^]^‐ this article aims to provide a comprehensive overview for both practitioners and materials scientists focused on the preparation of new biodegradable materials and the exploration of their potential applications. By categorizing applications across multiple domains and detailing each material's key characteristics, it offered a roadmap for the development and deployment of novel biodegradable materials. Moreover, the review assesses the advantages and practical challenges of different biodegradable materials and explores future directions, including degradability, preparation methods, structural processing, and application classification. By integrating perspectives from materials science, environmental engineering, and the circular economy, it emphasizes the role of biodegradable materials in transitioning from laboratory innovations to large‐scale, sustainable applications. Ultimately, the review aims to advance a zero‐waste society and promote environmentally friendly medical advancements.

## 2 Biodegradable Implants for Internal Medicine

Biodegradable implants are driving a seismic shift in internal‐medicine therapies by doing away with the very drawbacks that have long haunted permanent metal devices. Instead of leaving behind a lifetime of foreign material‐along with chronic inflammation, vessel restenosis, impaired vasomotor function, imaging artifacts, and the ever‐looming need for repeat procedures‐these ingenious constructions deliver full mechanical or electrical support only for as long as healing demands. Once their job is done, they dissolve seamlessly into harmless byproducts, leaving nothing but restored, healthy tissue in their wake. This “treat‐and‐vanish” philosophy transforms patient care at every turn. With no hardware left to remove or revise, the risks and costs associated with follow‐up surgeries all but disappear. Natural vessel tone and tissue integrity are preserved because nothing foreign remains to provoke dysfunction. Imaging studies become clearer than ever, free from the distortions that metallic struts once imposed on MRI, CT or ultrasound scans.^[^ ^58^ ^]^ Moreover, by precisely engineering the chemistry of bioresorbable polymers or metal alloys, clinicians can tailor how quickly an implant fades away‐whether it's a stent that supports a healing artery for just the right duration, a bone screw that relinquishes its hold as new tissue takes over, or a temporary pacing lead that quietly melts away after electrical stability returns. In effect, biodegradable implants don't merely enhance device therapy‐they redefine it, transforming once‐permanent fixtures into ephemeral allies on the road to lasting health.

### 2.1 Biodegradable Cardiovascular Implants

#### 2.1.1 Biodegradable Cardiac Pacing Devices

**Figure 1:**
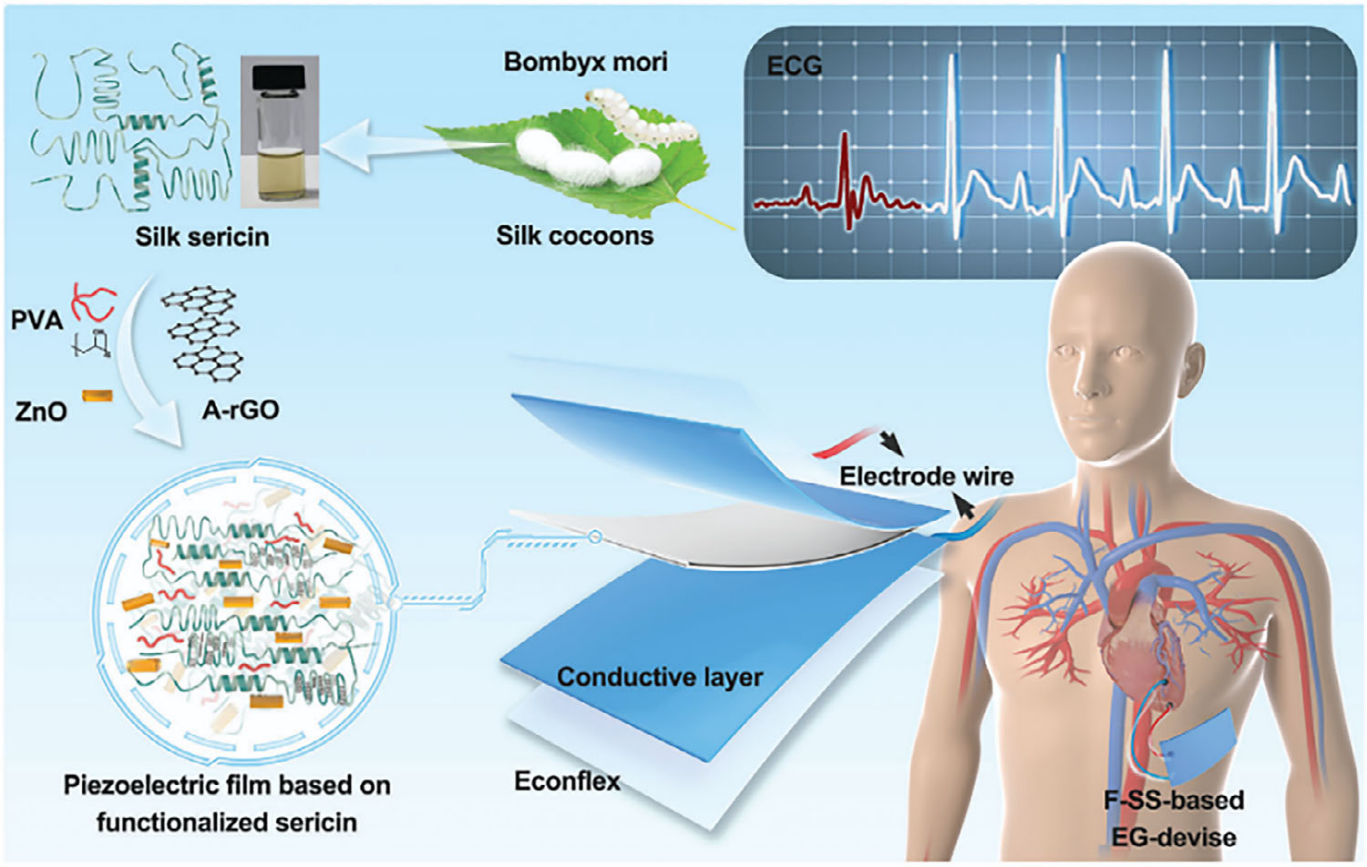
Scheme of functionalized SS and the potential application in CIEDs for its corresponding piezoelectric device (EG‐device). Adapte with permission. ^[^ ^59^ ^]^ Copyright 2025, Wiley‐VCH GmbH.

Cardiovascular medicine addresses prevention, diagnosis, and treatment of heart and vascular diseases, including coronary artery disease, heart failure, arrhythmias, hypertension, atherosclerosis, and aneurysms. Management spans lifestyle modification, pharmacotherapy, noninvasive and invasive diagnostics, interventional cardiology, catheter‐based procedures, vascular surgery, endovascular therapies, cardiac rehabilitation, and heart transplantation when needed. Cardiovascular disease remains the leading cause of heart failure and mortality, yet the scarcity of donor hearts severely limits transplantation. As a result, implantable cardiovascular devices have become the primary therapeutic option. Heart pacing devices are a very important treatment for cardiovascular disease. Conventional pacemakers are bulky, rigid devices with finite batteries requiring surgical implantation and revision, posing infection and trauma risks. Biodegradable materials offer a promising alternative for pacing. In the early stages of developing biodegradable pacing materials, naturally abundant polymers like silk sericin and chitosan first captivated researchers with their intrinsic biodegradability and ready availability. Silk sericin, a natural polymer rich in configurable asymmetric amino acids and endowed with notable bioactivities, has emerged as an ideal building block for an implantable, piezoelectric self‐powered device. After functionalization, the sericin film exhibited a longitudinal piezoelectric coefficient (*d_33_*) of 12 pC N^−1^ when integrated into a pacing module.^[^ ^59^ ^]^ The corresponding energy‐generating device (EG device) produced electrical output under mechanical deformation both in vitro and in vivo (**Figure** **1**). Remarkably, manual compression of the EG device yielded an instantaneous power density of 218.5 µW m^−2^, which is enough to reinitiate cardiac contractions and correct atrioventricular block in a preclinical model. Thanks to its excellent biocompatibility and physiological biodegradability, this functionalized silk sericin‐based EG device holds substantial promise for self‐powered cardiac implantable electronic devices (CIEDs) and other degradable implantable electronics.

Although natural polymers boast inherent biodegradability and widespread availability, their mechanical strength falls short of the rigorous demands of a pacing system. As a result, the development of biodegradable alloys and other high‐performance materials has become essential. A recent study developed flexible, self‐powered, photoelectric cardiac stimulators using a‐Si:H radial p‐i‐n junctions on Si nanowires atop aluminum foil.^[^ ^60^ ^]^ Under AM1.5G illumination, these conformal stimulators produce 0.67 V and 12.7 mA cm^−^^2^, powering heartbeats via a modulated 650 nm laser. In pigs, light‐triggered pacing increased rates from 101 to 128 bpm. By vacuum arc‐melting and rapid cooling via copper‐mold suction casting, a series of degradable temporary pacing electrode materials composed with FeMoCCu amorphous alloy can be synthesized.^[^ ^61^ ^]^ Potentiodynamic polarization measurements indicate that the Fe₆_3_Mo_2_₀C₁₇ amorphous composite corrodes at roughly 4.1 µA cm^−^^2^, a rate ascribed to its relatively high crystalline‐phase content and low charge‐transfer resistance. With its balanced properties and inherent biodegradability, this Fe‐based amorphous composite is a strong contender for use as a transient pacing‐electrode material. Given that cardiac pacing systems are generally designed for long‐term implantation, research has traditionally centered on their biocompatibility. As a result, the chief application of biodegradable devices in cardiology to date has been in vascular repair.

#### 2.1.2 Biodegradable Vascular Repairing Implants

Vascular diseases constitute a leading cause of mortality worldwide, necessitating the development of engineered materials that replicate the characteristics of natural vascular tissues within vascular tissue engineering (VTE). In contrast to the long‐term use of conventional pacing systems, interest in biodegradable implants has grown precisely because permanent devices carry inherent risks. Take vascular stents, for example. While traditional metal stents can immediately restore vessel patency, their permanent presence provokes chronic inflammation and neointimal hyperplasia, which in turn contribute to restenosis and in‐stent thrombosis. Beyond these biological responses, retained metal scaffolds also impede natural vasodilation and vasoconstriction, complicate imaging and follow‐up interventions, and elevate bleeding risk. Accordingly, natural proteins and polysaccharides have emerged as the materials of choice for biodegradable implant research. Recent studies have demonstrated that silk fibroin and alginate can be utilized for the biodegradable VTE. Silk fibroin (SF) has emerged as a promising biomaterial due to its exceptional biocompatibility and extensive applications in orthopedic and cardiovascular domains. However, the rapid degradation and inadequate mechanical properties of SF have limited its effectiveness in repairing large‐scale vascular defects. To address these challenges, magnetic silk fibroin scaffolds (MSFCs) have been developed, exhibiting enhanced mechanical strength, reduced degradation rates, and improved biocompatibility.^[^ ^37^ ^]^ In vitro and in vivo studies have demonstrated that the degradation rates of MSFCs are significantly reduced due to hydrogen bond formation between silk fibroin and MNPs, as well as tyrosine (Try) binding that inhibits the hydrolysis of internal iron atoms. Magnetic nanoparticles have also been shown to promote vascular endothelial cell growth, thereby improving scaffold biocompatibility and functionality. In addition to MSFCs, sulfated alginate‐based elastomers (BASPU) with a dual network that mimics heparin have been developed.^[^ ^38^ ^]^ These materials offer high toughness, mechanical properties akin to vascular tissue, self‐healing capabilities, and effective anticoagulant effects, making them ideal for tissue‐engineered vascular grafts. An ionic dual network, formed by the sulfate groups in sulfated alginate, provides exceptional toughness (up to 61 MJ m^−^^3^) and tunable mechanical properties, including a Young's modulus ranging from 0.9 to 6.6 MPa and tensile strength from 2.2 to 7 MPa. Additionally, poly (glycerol sebacate) (PGS) has been modified by incorporating palmitic acid to slow its degradation.^[^ ^42^ ^]^ Two variants, 9‐PPGS (9 % palmitate) and 16‐PPGS (16 % palmitate), were tested, with the 16‐PPGS grafts demonstrating superior performance. These grafts exhibited lumen sizes comparable to those of natural arteries, the development of an endothelial layer within four weeks, and reduced inflammation. After twelve weeks, the 16‐PPGS grafts displayed properties most like natural arteries, underscoring that controlling the degradation of PGS leads to improved graft performance.

**Figure 2:**
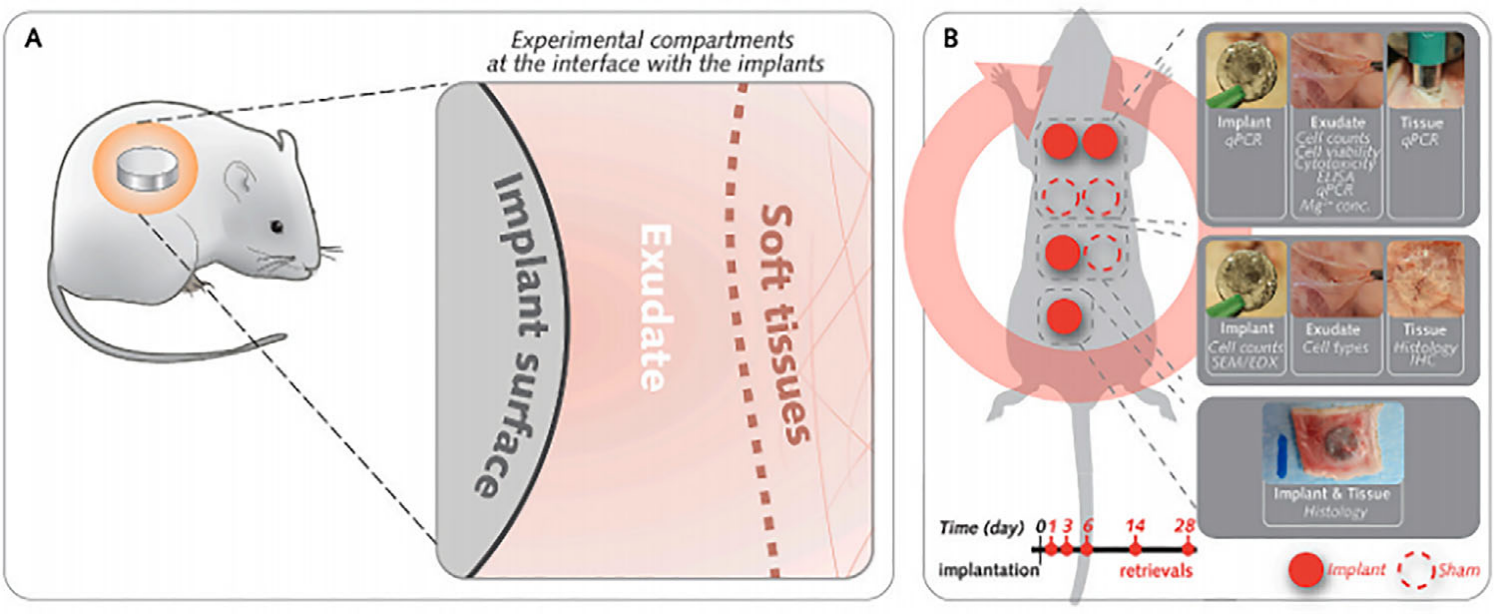
The rat subcutaneous model with the implants. Adapte with permission.^[^ ^22^ ^]^ Copyright 2023, The Authors.

However, in applications demanding high load‐bearing capacity and structural integrity, biodegradable metal alloys‐particularly magnesium and zinc‐based formulations‐remain the materials of choice. These alloys deliver the requisite mechanical strength and stiffness for short‐ to mid‐term support, while still degrading over time to eliminate permanent hardware. Further advancements have been investigated using a rat soft tissue model with an observation period ranging from 1 to 28 days to assess the surface properties of pure Mg implants and the impact of Mg^2^⁺ release on cell behavior.^[^ ^22^ ^]^ It was revealed that the degradation of Mg exacerbated initial inflammation, with degradation products peaking at three days. This was associated with the promotion of chemotaxis and the upregulation of immune markers, although cytotoxicity was not induced. Additionally, vascularization was enhanced, and VEGF expression was elevated. As the repair process progressed, calcium and phosphorus were enriched on the surface of the Mg implant, the Mg^2^⁺ concentration around the implant decreased, and the fibrous coating became thinner compared to that observed on Ti implants. These findings provided valuable insights into the design of Mg‐based implants (**Figure** **2**). Moreover, an Mg‐2Zn‐0.6Zr‐0.6 Nd alloy designed for biodegradable cardiovascular stents was fabricated using indirect extrusion.^[^ ^62^ ^]^ This alloy achieved a tensile yield strength of 269 MPa, an ultimate tensile strength of 298 MPa, and 25.6% elongation. Electrochemical tests demonstrated a lower corrosion rate in DMEM (0.03 mm/year) compared to Hank's solution (0.07 mm/year).

Unlike the focus on the traditional investigation strategy which followed as materials design, properties testing, and application in biodegradable implants, when bioprinting was incorporated into the field of biodegradable implants, researcher paid more attention to the applicability of bioinks. Bioprinting has been recognized as an advanced technique for the creation of complex 3D tissues; however, challenges remain with current bioinks and printing methods in printing elastic and vascularized tissues. A major hurdle lies in identifying bioinks that offer good printability, biocompatibility, biomimetic properties, and mechanical performance. The use of recombinant human proelastin as a bioink for printing complex soft tissues has been demonstrated in a study.^[^ ^63^ ^]^ Vascularized heart structures were bioprinted and subjected to both in vitro and in vivo testing, where endothelial barrier function and spontaneous beating‐key characteristics of cardiac tissue‐were exhibited by these constructs. Minimal inflammation was induced, and efficient biodegradation was achieved upon implantation in rats. Additionally, another study investigated the failure behavior of a 4D‐printed polylactic acid (PLA) shape‐memory scaffold under dynamic conditions following self‐deployment.^[^ ^54^ ^]^ It was shown that the compressive force of the scaffold ranged from 0.06 to 0.39 N mm^−1^, and the recovery rate ranged from 85.3% to 93.4%. After eight weeks of implantation at 60°C, the degradation rate of the scaffold was accelerated due to microstructural damage caused during deployment. Finite element analysis linked the relationship between scaffold damage, vascular injury, and deployment temperature, providing valuable insights for the reduction of healthcare risks.

### 2.2 Biodegradable Implants for In Vivo Drug Delivery

In vivo drug delivery remains a critical concern for both researchers and clinicians, and any strategy that eases the treatment burden on patients and healthcare providers is warmly welcomed. Today, starch is by far the most common delivery material, over 90 percent of all oral tablets use it as a filler. Yet in demanding scenarios, such as maximizing the efficacy and minimizing the systemic side effects of small‐molecule anticancer drugs, starch alone no longer suffices. To address these challenges, biodegradable implants have emerged as a powerful alternative. As with other implantable systems, natural polymers are the first choice thanks to their abundance, biocompatibility, and biodegradability. Synthetic biodegradable polymers complement them by providing the mechanical strength that many natural materials lack. Moreover, recent advances in nanotechnology, incorporating polymeric nanoparticles, nanogels, and lipid carriers into implant matrices, have enabled finely tuned, controlled drug release and precise targeting of diseased tissues.

#### 2.2.1 Biodegradable Chitosan‐Based Drug Delivery Implants

Like silk fibroin, chitosan is a natural polymer widely used in both medical applications and food preservation. Recently, chitosan (CS)‐based materials have garnered considerable attention in the field of drug delivery systems. For example, a localized CS nanoparticle (NP) system was engineered by modifying PLGA nanoparticles to encapsulate triamcinolone acetonide (TA).^[^ ^64^ ^]^ This modification resulted in extended drug release duration, enhanced thermal stability, and increased drug bioavailability. The optimized NPs, with diameters ranging from 334 ± 67.95 to 386 ± 15.14 nm and a polydispersity index (PDI) between 0.09 and 0.15, achieved TA encapsulation efficiencies of 55–57%. Additionally, improvements in mucosal adhesion and ocular barrier penetration were observed. Concurrently, zirconia (ZrO_2_), a bioceramic esteemed for its stability and biocompatibility, has been deemed suitable for orthopedic implants. However, traditional porous ZrO_2_ exhibits limited bioactivity and drug loading capacity.^[^ ^65^ ^]^ To overcome these limitations, a hierarchical porous ZrO_2_ with interconnected pores was fabricated, thereby enhancing drug loading capabilities and enabling sustained release. When ibuprofen (IBU) was utilized as the model drug, the modified ZrO_2_ demonstrated an increased drug load of 125.66 mg g^−1^, which was further elevated to 194.17 mg g^−1^ following a CS coating. Moreover, the CS coating regulated drug release, achieving a cumulative release of 93.7%. Building on these developments, hybrid microgels synthesized via the CS nanocomposite emulsion method have emerged as a prominent focus for oral drug delivery applications.^[^ ^39^ ^]^ These synthetic hybrid microgels, comprising chitosan, glycidyl methacrylate (GMACTS), and SiO_2_‐vinyltrimethoxysilane (SiO_2_‐VTS), exhibited larger diameters (18.7 ± 12.3 µm) compared to those without SiO_2_‐VTS. The microgels demonstrated rapid and substantial drug release for immediate pain relief and showcased pH‐responsive behavior, particularly under acidic conditions, thereby facilitating more controlled drug delivery.

#### 2.2.2 Biodegradable Synthetic Polymer‐Based Drug Delivery Implants

Because many naturals don't offer enough stiffness on their own, degradable synthetics are added to shore up mechanical performance. In tissue‐engineering construction, scaffold resorption must track new tissue formation, so being able to dial in the breakdown rate is fundamental. With that goal, catheters made from ciprofloxacin‐loaded zein blended with 2–32 % PVP have been systematically studied.^[^ ^43^ ^]^ By adjusting the PVP content, precise regulation of degradation and drug release rates were achieved, with higher PVP levels resulting in accelerated degradation and increased drug discharge. In vivo experiments, it was confirmed that catheters containing 32% PVP degraded more rapidly. In addition to these systems, a pH/redox dual‐responsive, biodegradable polyphosphazene nano‐prodrug with a high drug loading capacity of 56.4% has been developed.^[^ ^66^ ^]^ This prodrug is designed to release doxorubicin (DOX) under tumor‐specific conditions‐extracellular pH 6.8 and intracellular pH 5.0 in the presence of approximately 10 mm glutathione within endosomes and lysosomes‐while maintaining stability under normal physiological conditions. Vitro studies revealed potent cytotoxicity against cancer cells with minimal adverse effects on normal cells, highlighting its potential for targeted cancer therapy. Addressing the challenges associated with controlling the degradation rate and mechanical performance of polycaprolactone (PCL), a widely utilized biodegradable implant material, a novel approach involving tannic acid (TA) incorporation has been employed.^[^ ^67^ ^]^ The resultant PCL‐co‐TA composite displayed accelerated degradation and increased water content proportional to the TA concentration. The composite degraded more rapidly in neutral and basic environments, exhibiting surface erosion and significant mass loss over 90 days while preserving stable mechanical properties. Additionally, it provided sustained drug release for 40 days and demonstrated strong antimicrobial activity against *E. coli* and *S. aureus*.

### 2.3 Biodegradable Nerve Repairing Implants

Nerve repair encompasses assessment and reconstruction of damaged peripheral and central nerves via microsurgical methods (neurorrhaphy, grafting, transfers, conduits), neurolysis, and regenerative therapies, combined with postoperative rehabilitation to enhance axonal growth, reinnervation, and restoration of sensory and motor function. Peripheral nerve trauma impacts approximately 13–23 people per 100,000 each year and remains a significant internal clinical challenge.^[^ ^68^ ^]^ Severe injuries to these nerves are notoriously difficult to mend, and current repair strategies often yield suboptimal results, risking lasting deficits in motor, sensory, and autonomic functions.^[^ ^69^ ^]^ Traditional peripheral nerve interfaces, such as non‐degradable nerve cuffs, require later removal to prevent permanent device retention, which raises both infection risk and overall treatment costs.^[^ ^70^, ^71^ ^]^ Moreover, foreign‐body reactions frequently trigger fibrotic encapsulation around implants at injury sites, complicating potentially cause additional nerve damage.^[^ ^72^, ^73^ ^]^ In contrast, recent progress in bioresorbable materials and devices has opened new avenues.^[^ ^74^, ^75^ ^]^ Fully biodegradable nerve interfaces may thus eliminate the need for secondary surgeries while improving long‐term outcomes. Basically, synthetic polymers such as PLGA, PCL, and PLLA or the combination of synthetic polymers and natural polymers scaffolds are employed as essential materials in tissue engineering applications. Recent studies have investigated the effects of varying PLGA concentrations within chitosan‐based nerve conduits on peripheral nerve regeneration.^[^ ^44^ ^]^ An optimal PLGA concentration was found to significantly enhance nerve regeneration, resulting in improvements in nerve structure, reinnervation, conduction, and functional recovery. Additionally, the PLGA scaffold supported Schwann cell migration and maturation. The balanced degradation products of PLGA and chitosan contributed to reduced inflammation, fostering a more favorable environment for nerve regeneration.

Another study introduced an electroceutical platform that integrates a fully biodegradable conductive nerve conduit with a wireless electrical stimulator to optimize nerve regeneration.^[^ ^45^ ^]^ The nerve conduit, composed of molybdenum (Mo) microparticles and polycaprolactone (PCL), was designed to overcome challenges associated with non‐degradable implants, which can obstruct nerve pathways and necessitate surgical removal. The electrical and mechanical properties of the Mo/PCL conduits were optimized by varying the concentrations of Mo and tetraglycol lubricant. In vivo tests demonstrated that the combination of the Mo/PCL conduit with controlled electrical stimulation significantly accelerated axon regeneration in rats with extensive sciatic nerve defects compared to conduits without electrical stimulation. Moreover, a biodegradable neural interface was developed for wireless, real‐time monitoring and recovery of severe nerve damage.^[^ ^55^ ^]^ This novel interface, integrated with machine learning, was designed to analyze nerve recovery and detect the early formation of traumatic neuromas, thereby enabling timely intervention and improved outcomes. Its biodegradable properties eliminate the need for retrieval, reducing the risk of infection and secondary tissue damage. This study underscores the potential of bioresorbable, multifunctional neural interfaces for the early diagnosis and treatment of nerve injuries.

Over the last five years, research on bioresorbable implants in internal medicine has concentrated on reducing tissue damage from prolonged implantation and lowering both the risks and costs of follow‐up surgeries. Naturally derived polymers, such as silk sericin and chitosan, are inherently biocompatible, biodegradable, and nontoxic, but they fall short in mechanical strength. In contrast, synthetic materials like PLGA, PCL, and PLLA deliver superior mechanical performance yet depend on petrochemical feedstocks and often retain trace catalyst residues. Furthermore, their degradation mechanisms are not fully understood, and the potential release of microplastics poses an additional concern. Consequently, thorough evaluation of the true biodegradation profiles of these implants remains a critical priority.

## 3 Biodegradable Implants for Surgical Practicing

Biodegradable implants have become increasingly integral to modern surgical practice, offering temporary mechanical support that gradually degrades as tissue healing progresses. Common applications include bone fixation plates, soft‐tissue anchors, vascular stents, and wound‐closure devices. Synthetic polymers such as PLA, PGA) and their copolymers (PLGA, PCL) deliver tunable strength and degradation rates, while natural biopolymers like collagen, chitosan, and silk fibroin add inherent biocompatibility. By eliminating the need for secondary removal surgery, these implants reduce infection risk, patient discomfort, and overall healthcare costs. Key challenges remain precisely predicted in vivo degradation, ensuring consistent mechanical integrity under complex physiological loads, and fully characterizing the biological response to degradation by products.

### 3.1 Biodegradable Orthopedic Implants

Orthopedics encompasses the diagnosis and treatment of disorders affecting bones, joints, muscles, tendons, ligaments, and nerves, including congenital deformities, trauma, degenerative conditions, sports injuries, and spinal pathologies. Interventions range from rehabilitation and fracture care to arthroscopy and joint replacement, all aimed at restoring function, mobility, and quality of life. Orthopedic biodegradable implants play a pivotal role in musculoskeletal repair by offering temporary mechanical support that gradually degrades, eliminating the need for secondary removal and thereby reducing infection rates and surgical costs. By synchronizing degradation kinetics with bone healing, they avert stress shielding, encourage natural bone remodeling, and minimize chronic complications linked to permanent hardware. This innovation enhances patient comfort, accelerates rehabilitation, and eases healthcare burdens, making these implants indispensable for fracture fixation, ligament reconstruction, and pediatric orthopedics. Consequently, regenerative strategies, spanning tissue engineering, biomaterial scaffolds, and degradable implantable matrices, have emerged as core research areas. Emerging biomaterials fall into three main categories: bone‐compatible alloys (e.g., porous zinc scaffolds, biodegradable zinc), nanocomposites (e.g., self‐degrading “nano‐armor” coatings),^[^ ^46^ ^]^ and organic polymers (e.g., PLGA, PLA, PCL, hydrogels).^[^ ^76^ ^]^ Each class offers unique advantages, such as biocompatibility, tunable degradation rates, and robust mechanical integrity, positioning them as promising alternatives to conventional grafts.

#### 3.1.1 Organic Polymers‐Based Biodegradable Orthopedic Implants

##### Natural Polymer‐Based Biodegradable Orthopedic Implants

Natural polymers are indispensable in the development of biodegradable implants for both internal medicine and surgical practice. Silk fibroin (SF), a star biodegradable and biocompatible material broadly utilized in internal medicine, is suitable for osteochondral repair but lacks inherent bioactivity for cell adhesion and proliferation.^[^ ^77^ ^]^ To enhance its bioactivity, elastin‐like polypeptide (ELP) was incorporated into SF through a simple de‐hydrothermal treatment,^[^ ^34^ ^]^ thereby modifying and strengthening the scaffold structure. Bone mesenchymal stem cells (BMSCs) and chondrocytes exhibited improved spreading and growth on ELP‐modified scaffolds. Experimental results indicated that these modified scaffolds promoted superior bone and cartilage formation compared to unmodified SF scaffolds, highlighting their potential for clinical osteochondral repair. Further advancements in SF‐based materials include the development of a Silk fibroin (SF)‐based bioink for digital light processing (DLP) 3D bioprinting.^[^ ^78^ ^]^ Methacrylated SF (SF‐MA) achieved a methacrylation rate of 67.3 %, resulting in photocurable hydrogels with bone‐mimetic properties, such as a compressive modulus of 12–96 kPa and degradation rates between 48‐91% over 21 days. Encapsulated pre‐osteoblasts maintained high viability, while 15 % SF‐MA hydrogels supported cellular growth, proper morphology, and enhanced calcium deposition and osteogenesis, further augmented by induction factors.

##### Synthetic Polymer‐Based Biodegradable Orthopedic Implants

**Figure 3:**
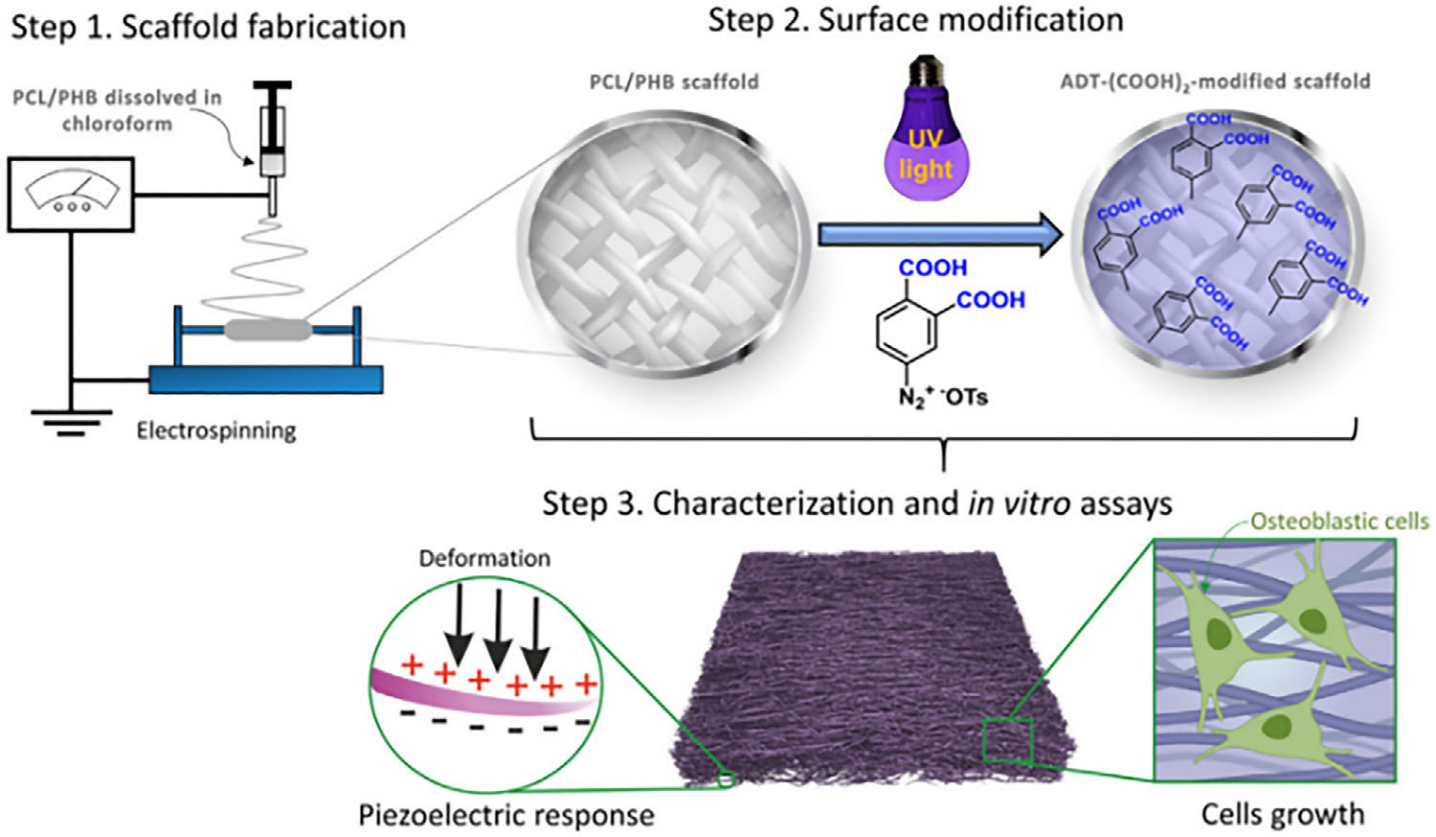
The fabrication of electro‐spun fibrous scaffolds. Adapte with permission.^[^ ^80^ ^]^ Copyright 2020, Elsevier Ltd.

Precisely tailored to complement natural polymers, synthetic variants offer enhanced mechanical strength alongside bespoke functional properties. For instance, CaCO_3_‐mineralized scaffolds were developed using poly (*R*)‐3‐hydroxybutyrate (PHB) and poly 3‐hydroxybutyrate‐co‐3‐hydroxyvalerate (PHBV).^[^ ^79^ ^]^ Mineralization was achieved through in situ synthesis of CaCO_3_ in vaterite and calcite forms via ultrasound. Comparative analysis indicated that porosity and surface charge significantly influenced mineralization under dynamic conditions. PHB scaffolds exhibited a 4.3‐fold increase in piezoelectric charge and approximately 15% higher porosity than PHBV, leading to more uniform CaCO_3_ deposition and a doubled mass. Post‐mineralization, scaffolds became super hydrophilic, enhancing apatite formation and osteoblast adhesion. Conversely, PHBV scaffolds with increased CaCO_3_ supported superior osteoblast growth, as validated by µCT and biocompatibility assays. By modifying them with 3,4‐dicarboxybenzenediazonium tosylate through ultraviolet light‐induced aryl radical formation, another novel method was developed to improve the wettability and cell spreading of piezoelectric polyhydroxybutyrate (PHB) and non‐piezoelectric polycaprolactone (PCL) scaffolds.^[^ ^80^ ^]^ This modification did not alter the scaffold structure but significantly reduced the water contact angle, enhancing hydrophilicity. Although the piezoelectric response of PHB scaffolds was slightly decreased, it remained intact. Osteoblastic cells exhibited better spreading, higher density, and the formation of distinct networks on the treated surfaces after seven days, indicating enhanced potential for bone tissue engineering applications (**Figure** **3**).

Citrate‐based materials address the challenge of creating bioactive materials with adjustable mechanical properties and degradation rates. Citrate‐based polymers combined with glycerophosphate salts, specifically β‐glycerophosphate disodium (b‐GP‐Na) and glycerophosphate calcium (GP‐Ca), were synthesized via a one‐pot reaction to form poly (octamethylene citrate glycerophosphate) (POC‐GP) polymers.^[^ ^56^ ^]^ These polymers exhibited high tensile strength and customizable mechanical properties. Notably, POC‐GP‐Ca demonstrated enhanced cell compatibility and promoted osteogenic differentiation of human stem cells in vitro. In a rabbit model, POC‐GP‐Ca/HA composites significantly improved bone regeneration, underscoring their potential for bone tissue engineering applications. Poly (lactic acid) (PLA) is another biodegradable and biocompatible polymer with significant promise in bone tissue engineering. However, its slow degradation rate limits effective bone regeneration. Studies investigating PLA scaffold degradation mechanisms‐such as autocatalysis, chain scission, and surface and bulk degradation‐highlight the roles of crystallinity, molecular weight, and pH.^[^ ^81^ ^]^ Methods to accelerate PLA degradation include blending, copolymerization, and surface modification. Additionally, rapid prototyping technologies have been explored to optimize scaffold architecture, while the potential of 4D printing to enhance PLA scaffold functionalities has been examined.

Polyurethane (PU) membranes have also been explored for their biodegradability and biocompatibility. AT‐based polyurethane (FPAT) membranes were created using electrospinning, inspired by the bone tissue's natural electric field, to promote osteogenesis.^[^ ^57^ ^]^ Comprehensive studies demonstrated that FPAT membranes influence macrophages and mesenchymal stem cells (MSCs). They promote the M2 macrophage phenotype, reduce ROS levels, and enhance MSC proliferation and osteogenic differentiation. Histological analyses and gene expression studies in a rat bone defect model confirmed the membranes' roles in immunomodulation and bone healing, highlighting their potential in bone regeneration and evaluation of conductive materials. Additionally, a 3D‐printable, biodegradable polyurethane (WBPU) modified with amino acids was developed via a water‐based green chemistry process.^[^ ^82^ ^]^ WBPU offers enhanced flexibility, improving tissue compatibility during implantation and minimizing damage to surrounding tissues. Histocompatibility assessments revealed that WBPU does not induce acute rejection or inflammation in vivo. Fabricated at low temperatures (50–70 °C), WBPU scaffolds supported chondrocyte and fibroblast adhesion and growth. Moreover, porous gelatin/hydroxyapatite (G/H) scaffolds combined with human placenta‐extracted hPE also exhibit improved mechanical properties and bioactivity.^[^ ^35^ ^]^ In vitro studies demonstrated that G/H/hPE scaffolds significantly increased cell proliferation (G/H: 351.1 ± 13.3% vs. G/H/hPE: 430.9 ± 8.7% at day 14) and upregulated osteogenic markers (ALP: 3.4‐fold; Runx2: 3.9‐fold; BMP2: 1.7‐fold; OPN: 2.4‐fold; OCN: 4.8‐fold at day 21) in human adipose‐derived stem cells (hASCs) compared to G/H alone.

#### 3.1.2 Alloy‐Based Biodegradable Orthopedic Implants

**Figure 4:**
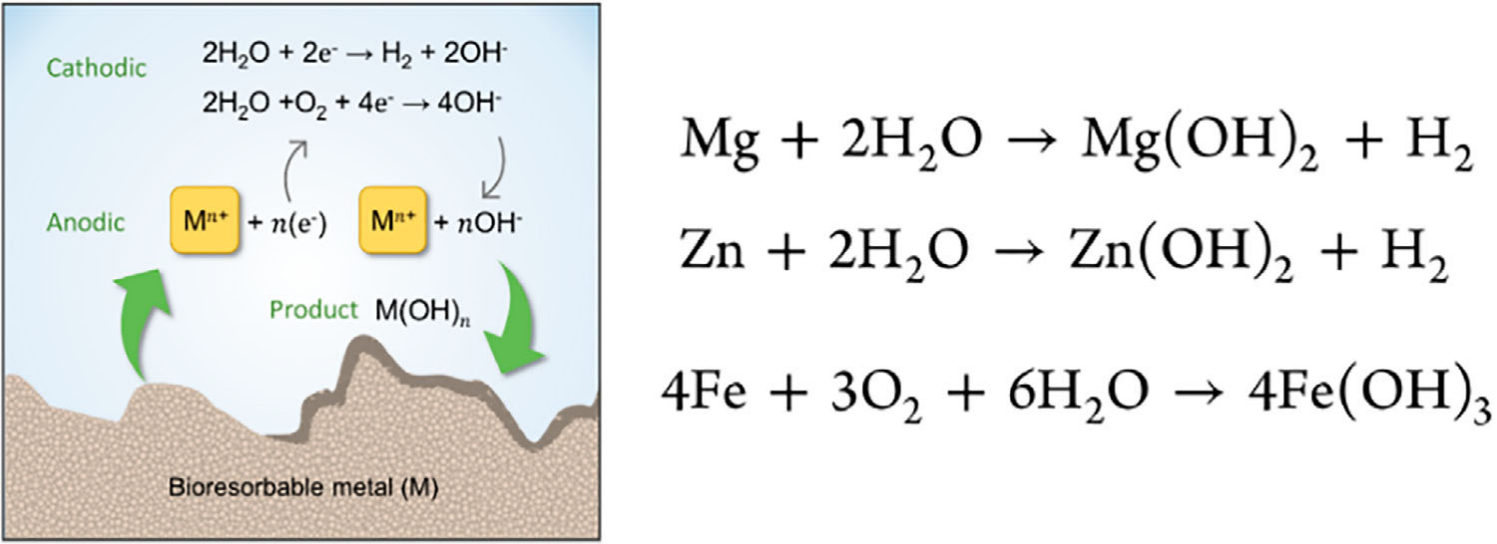
Chemical reactions of biodegradable alloys. Adapte with permission.^[^ ^75^ ^]^ Copyright 2023, American Chemical Society.

Alloy‐based biodegradable orthopedic implants (e.g., Mg, Zn, Fe) have become indispensable because they significantly outperform organic‐based materials in both load‐bearing capacity and predictable resorption. In contrast to polymeric devices, which often fall short in rigidity and display erratic degradation behavior, magnesium and zinc alloys match the stiffness of cortical bone and allow fine tuning of their corrosion kinetics. Their metallic framework ensures continuous mechanical support throughout the healing phase while mitigating inflammatory responses. Organic implants, by comparison, degrade via hydrolysis and generate a range of byproducts that compromise their application in weight‐bearing sites. Consequently, alloy systems deliver the combination of strength and controlled dissolution essential for reliable bone repair. The most widely studied biodegradable metals include alkaline‐earth elements (for example, Mg and Ca), transition metals (such as Zn, and Fe), and their various alloys.^[^ ^75^ ^]^ These materials can be processed into ultrathin films, foils, powders, wires, or embedded within bioresorbable polymer matrices. Their corrosion proceeds through coupled anodic and cathodic reactions, producing a complex mixture of degradation products. Upon immersion in water or physiological fluids, the metal (M) is oxidized to Mⁿ⁺ ions and electrons (anodic reaction), while the freed electrons facilitate reduction reactions that generate hydroxide ions, hydrogen gas, metal hydroxides, and phosphate species (cathodic reaction) (**Figure** **4**).

##### Zinc‐based biodegradable orthopedic implants

The advancement of temporary biomedical implants that obviate the necessity for subsequent removal surgeries is a critical area of research. Among biodegradable metals, magnesium (Mg) and zinc (Zn), along with their alloys, stand out as promising candidates for orthopedic applications due to their favorable biodegradability and biocompatibility profiles.^[^ ^47^, ^83^ ^]^ Zn‐based alloys offer improved corrosion control compared to pure Zn; however, their mechanical strength remains insufficient for load‐bearing applications. To overcome this limitation, ternary Zn‐1Fe‐xSr (x = 0.5, 1, 1.5, 2 wt.%) alloys were synthesized via stir casting.^[^ ^84^ ^]^ The incorporation of strontium (Sr) facilitated the formation of the SrZn_13_ phase and refined the grain structure, resulting in a 55.7% increase in ultimate tensile strength and a 58.4% enhancement in hardness for Zn‐1Fe‐2Sr compared to Zn‐1Fe. Nevertheless, higher Sr concentrations also led to increased corrosion current density and elevated degradation rates, which subsequently decreased with prolonged immersion periods. Advancements in additive manufacturing, particularly laser powder bed fusion (L‐PBF), have enabled the fabrication of pure Zn porous scaffolds with diamond lattice structures tailored for orthopedic use.^[^ ^23^ ^]^ These scaffolds exhibit compressive strengths and rigidity comparable to cancellous bone, alongside degradation rates that support bone regeneration. In vivo evaluations using a rabbit femur defect model over 24 weeks demonstrated excellent biocompatibility and osteogenic potential, with no adverse responses observed in vital organs.

Further optimization efforts have focused on porous Zn‐Li‐Ca scaffolds with 70 % porosity, fabricated using NaCl porogens.^[^ ^85^ ^]^ The Zn‐0.8Li‐0.8Ca variant exhibited significantly enhanced mechanical properties (yield strength: 35.80 ± 1.89 MPa; elastic modulus: 2.45 ± 0.19 GPa) and controlled degradation rates (7.51% weight loss; 0.21 mm/year) in simulated body fluids. The addition of calcium (Ca) accelerated degradation by compromising the protective Li_2_CO_3_ layer, highlighting the scaffold's potential for bone repair. Moreover, alloying Zn with transition metals such as vanadium (V), chromium (Cr), and zirconium (Zr) has been shown to enhance mechanical strength and reduce localized degradation, as demonstrated in vivo rat studies involving subcutaneous, aortic, and femoral implantations.^[^ ^30^ ^]^ Surface modifications further augment the biocompatibility and osteogenic capacity of Zn‐based alloys. Composite coatings composed of calcium phosphate (CaP) and polylactic acid (PLA) applied to Zn‐Mn‐Mg alloys via hydrothermal and dip‐coating techniques enhanced corrosion resistance and promoted CaP deposition.^[^ ^86^ ^]^ Notably, the hydrothermal/PLA (12 w/v) layer exhibited superior cytocompatibility, significantly improving MC3T3‐E1 cell survival and attachment, thereby reinforcing the potential of zinc‐based materials for degradable orthopedic implants.

Post‐processing treatments, particularly heat treatments of rapidly corroding, degradable metals, are well established for suppressing aggressive degradation and in vivo hydrogen evolution via surface passivation. To improve outcomes in infected bone repair, a new approach combined copper alloying with post‐annealing in laser powder bed fusion fabricated zinc implants.^[^ ^87^ ^]^ The heat‐treated Zn‐2Cu alloy attained a yield strength of 203 MPa through synergistic strengthening, while the timed release of Zn^2^⁺ and Cu^2^⁺ at therapeutic levels delivered dual bio‐functional effects in vitro and in vivo. In a related study, two Zn‐Cu alloys (0.8 and 1.5 wt.% Cu) were evaluated for hemocompatibility and biocompatibility in their as‐fabricated and annealed states.^[^ ^88^ ^]^ The 1.5 wt.% Cu alloy initially contained roughly twice as many Cu‐Zn_5_ precipitates as the 0.8 wt.% variant; annealing partially dissolved these second‐phase particles. After six months of implantation in rat arteries, the as fabricated 1.5 wt.% Cu alloy exhibited the least neointimal thickening and smooth muscle infiltration, despite a slight uptick in inflammatory markers. By contrast, heat treatment aggravated vascular responses, producing thicker neointima, greater smooth muscle cell presence, and small necrotic zones. In vivo corrosion proceeded mainly by pitting, most severe in the annealed alloys, whereas the as‐fabricated 1.5 wt.% Cu alloy corroded more uniformly. These variations are attributed to heat‐induced microstructural alterations that favor localized attack.

##### Magnesium‐Based Biodegradable Orthopedic Implants

**Figure 5:**
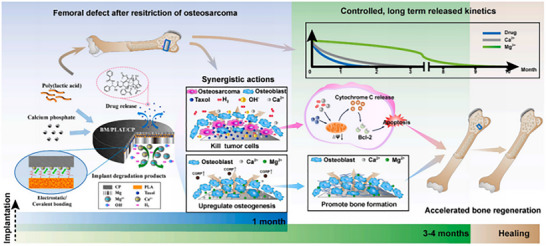
Schematic diagram of BM/PLAT/CP implant design and bio‐application in treating tumor‐induced bone defects. Adapte with permission.^[^ ^33^ ^]^ Copyright 2023, The Authors.

Mg‐based alloys have gained prominence as essential materials for orthopedic implants, particularly in managing tumor‐induced bone defects, which are associated with significant side effects, elevated risks of tumor recurrence, and prolonged healing periods. Traditionally, magnesium alloys have been utilized primarily for their favorable mechanical properties, limiting their application to structural support within orthopedic contexts. However, recent advancements have facilitated the development of magnesium‐based composite implants incorporating calcium‐based coatings, enabling controlled degradation and the localized delivery of chemotherapeutic agents such as Taxol.^[^ ^33^ ^]^ The controlled degradation of magnesium in aqueous environments serves a dual purpose: it promotes osteogenesis and modulates the tumor microenvironment through the release of degradation byproducts. Specifically, the implants release hydrogen (H_2_) and calcium ions (Ca^2+^), which, in conjunction with the localized release of Taxol, activate cellular pathways that induce cytotoxicity in tumor cells and inhibit their proliferation. This targeted chemotherapeutic approach not only mitigates systemic toxicity but also enhances bone repair and regeneration, as demonstrated in vivo rat models. Consequently, this multifaceted strategy represents a promising solution for treating tumor‐related bone defects by integrating mechanical support with therapeutic functionality in biodegradable magnesium‐based implants (**Figure** **5**).

Additionally, biodegradable Mg‐based implants have been explored to facilitate osteogenesis in osteoporotic conditions, addressing the prevalent magnesium deficiency associated with osteoporosis. A Mg‐Zn‐Ca alloy (ZX00) implant was evaluated in ovariectomy‐induced osteoporotic (Osteo), old healthy (OH), and juvenile healthy (JH) female rat models using in vivo micro‐computed tomography (µCT).^[^ ^26^ ^]^ After eight weeks, the Osteo cohort exhibited a marked reduction in trabecular bone volume, accelerated implant degradation, and increased gas formation relative to the OH and JH groups. Moreover, a subset of osteoporotic rats developed sclerotic rims, potentially attenuating foreign‐body responses and preventing osteonecrosis. μ‐CT analyses corroborated diminished bone volume in the Osteo group, while histological examinations revealed enhanced implant degradation in osteoporotic subjects. Previous investigations evaluated the biocompatibility and degradation kinetics of magnesium hydroxide and RS66, a robust ZK60‐based alloy, within rabbit condyles using micro‐CT and histological methodologies.^[^ ^29^ ^]^ Both materials significantly promoted osteogenesis through distinct mechanisms: RS66 primarily stimulated periosteal bone formation on the medial condylar surface, whereas magnesium hydroxide favored cancellous bone proliferation. Periosteal bone formation concluded at six and eight weeks, respectively. These findings substantiate prior reports indicating that magnesium augments bone growth, potentially through specific osteogenic signaling pathways.

##### Zinc–Magnesium Alloy Biodegradable Orthopedic Implants

Zn‐based alloys have emerged as promising biodegradable materials for orthopedic applications due to their favorable biodegradability and biocompatibility. Nevertheless, pure Zn lacks the mechanical strength required for load‐bearing implants. To address this, a Zn‐3Mg‐0.7Mg_2_ Si composite was synthesized via high‐pressure solidification,^[^ ^84^ ^]^ exhibiting a homogeneous distribution of fine MgZneji70012 precipitates within an α‐Zn matrix. This composite demonstrated a yield strength of 406.2 MPa, an ultimate compressive strength of 1181.2 MPa, and the capacity to endure up to 60% strain without fracturing. Corrosion assessments revealed a corrosion potential of ‐0.930 V, a current density of 3.5 µA cm^−^^2^, and a corrosion rate of 46.2 µm/year. Immersion in Hanks' solution showed degradation rates of 42.8 µm/year after one month and 37.8 µm/year after three months. Additionally, cytocompatibility assays indicated superior cell viability compared to pure Zn and other composites at concentrations ≤25%, underscoring its potential for orthopedic applications. Further research has concentrated on binary Zn alloys incorporating elements such as Mg, Ca, Sr, Li, Mn, Fe, Cu, and Ag to enhance their mechanical and biological performance for bone implants.^[^ ^27^ ^]^ While magnesium‐based biodegradable metals typically exhibit lower strength compared to biodegradable polymers, the incorporation of lithium (Li) and Mg significantly reinforces Zn alloys. Alloying Zn with Mg, Ca, Sr, and Li improves cytocompatibility, osteogenic potential, and osseointegration. For instance, optimized Zn‐Li alloys, such as Zn‐0.8Li‐0.4Mg and Zn‐0.8Li‐0.8Mn, demonstrated tensile strengths of 646.69 MPa and elongations of 103.27%, respectively, rendering them viable candidates for load‐bearing orthopedic applications with mechanical strengths comparable to pure titanium.

To synergize the advantageous properties of Zn and Mg alloys, Zn/Mg bimetallic composites have been developed.^[^ ^89^ ^]^ Zn/Mg multilayered composites fabricated via roll bonding exhibited progressively thinner layers and increased interfaces with additional bonding cycles. After 14 cycles, the composite achieved a yield strength of 411 ± 3 MPa and a tensile strength of 501 ± 3 MPa, surpassing single‐layer Zn/Zn or Mg/Mg counterparts. Post‐annealing at 150°C for 10 min reduced the yield strength to 349 ± 3 MPa while increasing elongation from 8% to 28%. Degradation rates in Hanks' solution ranged from 127 ± 18 to 6 ± 1 µm/year, demonstrating controlled biodegradation. These composites also exhibited excellent cytocompatibility, with over 100% cell viability in MG‐63 cells after three days. Enhancements through polymer/ceramic hybrid coatings have further improved Zn‐Mg alloys. Coating Mg‐Zn‐Ca alloys with hydroxyapatite (HA), NaOH treatment, and polycaprolactone (PCL) via dip‐coating markedly improved mechanical properties, adhesion strength, and corrosion resistance, reducing corrosion rates by 900‐fold compared to untreated alloys and tenfold relative to HA‐only coatings.^[^ ^90^ ^]^ Additionally, Mg6Zn alloys modified with Casingle‐bond pre‐coatings and PCL/HA/ZnO nanoparticle coatings exhibited fivefold bond strength improvements, increased surface roughness for enhanced cell attachment, controlled degradation, and over 90% antibacterial activity and cell proliferation.^[^ ^91^ ^]^ Moreover, super‐hydrophobic coatings composed of magnesium compounds and zinc, fabricated using a wet chemical method, achieved a water contact angle of 162.04°, enhancing corrosion resistance, inhibiting blood platelet adhesion, and demonstrating low hemolysis and high cell viability.^[^ ^92^ ^]^ These advancements significantly contribute to the development of Zn‐Mg alloys for medical implant applications. In summary, advancements in Zn, Mg, and Zn‐Mg alloy compositions, coupled with innovative surface modification techniques, have substantially enhanced the mechanical properties, corrosion resistance, and biocompatibility of biodegradable implants. These developments underscore the potential of alloy‐based biodegradable materials as robust candidates for a wide range of orthopedic applications.

##### Iron‐Based Biodegradable Orthopedic Implants

Materials for bone repair and fixation must endure significant mechanical loads temporarily and, ideally, biodegrade as they are replaced by new host bone. Iron is essential yet toxic at serum levels above 350‐500 µg dl^−^ and supports hemoglobin, myoglobin, and numerous enzymes, driving oxygen transport, ribonucleotide reduction, lipid metabolism, and protein/DNA repair.^[^ ^93^ ^]^ Thus, iron‐based biodegradable implants, which offered high yield strength, elastic modulus, and ductility, were broadly utilized in orthopedic practice. For instance, selective laser melting (SLM) of Fe‐Mn yields hierarchical scaffolds that retain load‐bearing strength after 28 days in simulated body fluid and exhibit higher corrosion rates than bulk iron.^[^ ^31^ ^]^ In vivo, the implantation into bone defects showed effective osseointegration and new bone formation after four weeks. To adjust degradation, Cu‐added Fe‐Mn alloys were developed.^[^ ^32^ ^]^ Static immersion and electrochemical polarization tests showed that higher Cu content accelerates corrosion, while Fe‐Mn‐Cu extracts displayed enhanced bactericidal activity against E. coli. MG63 and MC3T3‐E1 cytocompatibility assays revealed progressively increased cell proliferation for all formulations. Implantation in rabbit femurs produced no periprosthetic tissue necrosis. These SLM‐fabricated Fe‐Mn and Cu‐alloyed variants are thus promising, customizable, biodegradable scaffolds for load‐bearing bone repair. Repairing extensive bone defects continues to pose a major obstacle in tissue engineering. As a result, there is an urgent demand for scaffold materials that faithfully mimic both the mechanical strength and open‐porous microarchitecture of human cancellous bone, while maintaining excellent biocompatibility. By metal‐powder sintering, a cylindrical, phosphorus‐alloyed cellular scaffold was produced.^[^ ^24^ ^]^ In a sheep model of proximal tibial cancellous bone replacement, a new bone infiltrated the implant's pores and formed well‐mineralized tissue at the bone‐implant interface after 12 months. However, the study was limited by the scaffold's slow degradation, which was caused by occluding cavities that hindered the penetration and maturation of newly formed bones.

Although extensive in vitro and in vivo studies on cardiovascular stents have demonstrated that pure iron causes neither local nor systemic toxicity, research into local and systemic inflammatory responses following the implantation of porous, degradable bone substitutes remains insufficient. A recent study, which utilized a sheep model for evaluation of inflammation of novel iron‐based degradable bone implants, strengthened the safety of iron‐based degradable bone implants.^[^ ^25^ ^]^ Findings showed that histopathological examination of the brain, parenchymatous organs, implantation sites, and nearby lymph nodes revealed no marked inflammatory alterations versus controls. Elevated iron deposits were observed only in the soft tissues surrounding the implant and, to a lesser extent, within the popliteal lymph node. Neither total nor differential blood counts indicated an acute or chronic inflammatory response linked to the implant. Although ferritin and phosphorus levels rose slightly in the implanted group relative to preoperative baselines, all measurements stayed within sheep‐specific reference limits.

##### Other Alloy Biodegradable Orthopedic Implants

With the increasing prevalence of spinal diseases and the complexity of spinal surgeries, selecting appropriate implants is critical due to the spine's unique mechanical demands. Nitinol (NiTi) alloy, celebrated for its super elasticity, high strength, and fatigue resistance, presents as a viable candidate for spinal implant applications.^[^ ^94^ ^]^ NiTi lumbar‐like samples were fabricated using sintering and mechanical braiding techniques, resulting in materials that demonstrated an elastic modulus of 3.5 GPa and a compressive strength of 200 MPa at 6% strain. The alloy's thermoelastic properties closely match those of bone tissue, enhancing its suitability for orthopedic applications. Biocompatibility assessments revealed over 4×10^4^ viable cells after 48 hours, and its thermal properties are compatible with body temperature. Additionally, NiTi exhibited a corrosion resistance of approximately 0.3 Ω·cm^2^, further affirming its potential as a spinal implant. Furthermore, iron‐gallium (Fe‐Ga) alloys offer mechanical advantages and low saturation magnetization, although their magnetostrictive performance is limited by grain orientation.^[^ ^95^ ^]^ The Fe_81_Ga_19_ alloy, fabricated via selective laser melting (SLM) with various scanning paths, achieved optimal grain orientation when processed with a zigzag scanning pattern. This configuration resulted in a magnetostrictive ratio of 77.2 ppm, representing an increase of 23.9 % and 25.1 % compared to unidirectional and annular scanning paths, respectively. The alloy also demonstrated a high compressive strength of 448.6 MPa. Additionally, Fe‐Ga alloys exhibited favorable phase transition temperatures and corrosion resistance, making them promising candidates for spinal implant applications.

#### 3.1.3 Nano Formation‐Based Biodegradable Orthopedic Implants

Nano‐formulated biodegradable implants are transforming orthopedic care by integrating finely tuned nanoscale drug carriers within resorbable scaffolds. This design delivers growth factors, antibiotics, or anti‐inflammatory agents directly to injury sites for sustained therapy as the implant gradually degrades, obviating removal procedures. Their nanoscale topography enhances cell adhesion, proliferation, and osteointegration, accelerating bone regeneration and reducing aseptic loosening. Moreover, nanocomposite engineering permits precise tuning of degradation and mechanical strength, facilitating patient‐specific solutions. By uniting structural support with controlled, localized drug delivery, these implants offer unparalleled efficacy, positioning them as essential tools for advanced orthopedic interventions. However, scaling up uniform nano system production remains complex and costly, while uncontrolled burst release or unpredictable degradation by products can provoke local inflammation. Thus, the field is advancing toward multifunctional “smart” implants featuring stimuli‐responsive release, 3D‐printed architectures customized to individual anatomy. Innovations in bioinspired materials and AI‐driven design promise to elevate the safety, efficacy, and personalization of next‐generation orthopedic therapies.

##### Biodegradable Orthopedic Nano Scaffold

A significant challenge continues to be posed by the reconstruction of large bone defects in modern medicine. In orthopedic treatments, bone repair is accelerated by electrical stimulation, yet the direct integration of this cue into tissue‐engineered grafts has been hindered by technical difficulties. To address this, a fully biodegradable piezoelectric scaffold composed of poly (L‐lactic acid) nanofibers has been developed, which, when actuated by externally applied ultrasound, generates localized surface charges.^[^ ^53^ ^]^ By supplying the electrical signals required for osteogenesis, the need for high‐dose growth factors is obviated. In vitro, osteogenic differentiation of multiple stem cell types is significantly enhanced by ultrasound‐triggered piezoelectric activation. In vivo, robust new bone formation is achieved following implantation into critical‐sized calvarial defects. As a battery‐free, remotely controlled stimulator, this technology is regarded as a major leap forward in next‐generation bone graft substitutes. In a separate study, composite scaffold for guided bone regeneration were fabricated by grafting polycaprolactone onto chitosan via copper(I)‐catalyzed azide‐alkyne cycloaddition (CuAAC).^[^ ^96^ ^]^ To boost bioactivity, magnesium‐substituted hydroxyapatite (Mg‐HA) was incorporated into the scaffold matrix. Cytotoxicity testing by MTT assay confirmed the absence of significant cell death. Compared with simple CS/PCL/Mg‐HA blends, the CuAAC‐derived CS‐g‐PCL/Mg‐HA nanofibers demonstrated superior antibacterial efficacy, enhanced mechanical strength, and improved cell attachment. Moreover, elevated alkaline phosphatase activity and more intense Alizarin Red S staining revealed that the introduction of triazole linkages markedly promoted bone mineralization.

##### Biodegradable Orthopedic Nanofiber

**Figure 6:**
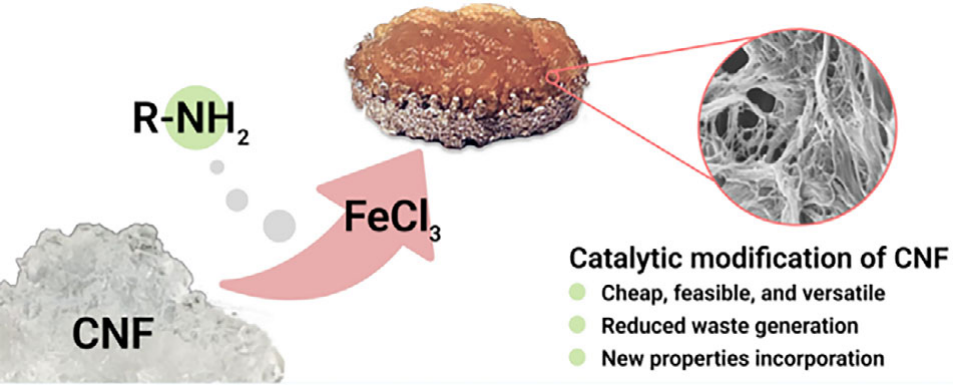
Functionalize cellulose nanofibrils by conjugating amine polymers. Adapte with permission.^[^ ^97^ ^]^ Copyright 2024, The Authors.

A recent investigation utilized FeCl_3_ Lewis acid catalysis to functionalize cellulose nanofibrils (CNFs) by conjugating amine polymers, including polyethyleneimine and Jeffamine, to carboxymethylated CNFs (c‐CNF).^[^ ^97^ ^]^ This functionalization process enhanced both the adhesiveness and thermal stability of the CNF materials. Mechanistically, the oxygen atoms inherent to cellulose stabilize the transition state during the functionalization reaction. A CNF‐PEI800 composite was synthesized, demonstrating considerable promise as a coating for medical implants. The resultant hydrogel exhibited both adhesive and antibacterial properties while concurrently supporting the proliferation and differentiation of human osteoblasts over a seven‐day incubation period (**Figure** **6**). Bionic coatings are critical for the efficacious healing of bone fractures; however, the post‐traumatic milieu, characterized by hypoxia, acidosis, and oxidative stress, can undermine their functionality. To address these impediments, a H‐Si@HA composite coating was engineered by integrating H‐silicene nanosheets (H‐Si) with a hydroxyapatite (HA)‐coated metal implant.^[^ ^48^ ^]^ This composite coating maintains stability under acidic conditions and attenuates oxidative stress by emulating enzymatic activities. Additionally, it confers cellular protection by inducing autophagy. As H‐Si undergoes degradation, it releases Si^4+^ ions in neutral environments, thereby promoting fracture healing through the upregulation of osteogenic gene expression in bone marrow‐derived stem cells (BMSCs) and superoxide dismutase 1 (SOD1) expression in osteoprogenitor cells. Transcriptomic analyses revealed that H‐Si@HA facilitates osteogenesis by modulating the expression of key genes implicated in bone formation. This self‐degradable coating exhibits substantial promise for enhancing fracture healing outcomes.

#### 3.1.4 With Potential Orthopedic Applicating Value's Biodegradable Implants

Biodegradable implants have attracted extensive interest in orthopedics because they provide temporary mechanical support and then resorb, eliminating secondary‐removal surgeries and thereby reducing infection risk and healthcare costs. While a serial of studies has established bona fide orthopedic models, many others have evaluated the potential of diverse biodegradable materials for orthopedic applications. The advancement of degradable bone implants is critical for enhancing bone regeneration strategies. Recent studies have leveraged material–structure–function integrated additive manufacturing (MSFI‐AM) to synthesize zinc‐based Zn‐Mg‐Cu alloys.^[^ ^28^ ^]^ This innovative approach has significantly improved the mechanical properties of the alloys and facilitated self‐strengthening during the degradation process. In vitro evaluations demonstrated that these alloys effectively promote osteogenesis, modulate immune responses, support angiogenesis, and exhibit antimicrobial properties. Additionally, investigations into Zn‐Mg alloys containing 0.68 wt.% Mg, extruded at 200°C, revealed enhanced mechanical performance and reduced corrosion rates upon modification with poly (methyl methacrylate) (PMMA), further validating their suitability for orthopedic implant use.^[^ ^98^ ^]^ In the domain of tissue engineering, understanding the interactions between cells and scaffolds is paramount. Chitosan‐gelatin‐poly (*L*‐lactic acid) double‐layer scaffolds fabricated via electrospinning have been shown to effectively induce the differentiation of airway epithelial cells into ciliated and goblet cells, highlighting their potential in facilitating tissue formation.^[^ ^99^ ^]^ The incorporation of self‐healing coatings represents a significant advancement for biodegradable implants, particularly magnesium alloys. A novel fluoride/poly (lactic acid)/amorphous calcium phosphate (F/P/ACP) coating, developed through chemical reaction deposition, functions both as a protective barrier and a self‐healing mechanism activated by pH fluctuations resulting from corrosion.^[^ ^100^ ^]^ This multifunctional coating markedly enhances the corrosion resistance and longevity of magnesium‐based implants without the need for additional inhibitors.

Infection remains a leading cause of implant failure, necessitating the development of effective antibacterial coatings. Traditional coatings often fail to prevent bacterial adhesion or merely immobilize dead bacteria, thereby reducing their antibacterial efficacy. To address this, a copper‐doped poly (3‐sulfopropyl methacrylate potassium salt) (Cu@PSPMAK) coating was synthesized, exhibiting both antibiofouling and bactericidal properties against *Staphylococcus aureus* and *Escherichia coli*.^[^ ^49^ ^]^ This coating maintains excellent biocompatibility, presenting a promising solution for preventing implant‐associated infections. Furthermore, biodegradable elastomers exhibit substantial promise in biomedical applications. High‐performance polyester elastomers with enhanced mechanical strength, elasticity, and self‐healing capabilities were developed by adjusting molecular weight, UPy content, and crystallization parameters.^[^ ^101^ ^]^ Additionally, elastomers synthesized through chelation, such as Fe^3^⁺‐crosslinked foams, demonstrated superior tissue compatibility and minimal fibrotic response in murine models, underscoring the potential of chelation‐based designs for novel biodegradable materials.^[^ ^102^ ^]^ In summary, significant advancements in zinc‐based alloys, self‐healing and antibacterial coatings, and biodegradable elastomers have markedly improved the mechanical integrity, biocompatibility, and functional performance of biodegradable implants. These developments enhance the clinical applicability of such implants in tissue regeneration and repair, offering promising avenues for future biomedical engineering innovations.

### 3.2 Biodegradable Implants for Wound‐Healing

Wound healing proceeds through four overlapping phases, hemostasis, inflammation, proliferation, and remodeling, to restore tissue after injury. These stages involve clot formation, immune response, new blood vessel growth, extracellular matrix deposition, re‐epithelialization, and scar maturation, all modulated by nutrition, oxygenation, infection status, and comorbidities. Biodegradable implants are indispensable in wound healing, providing temporary scaffolds that orchestrate tissue regeneration and then safely resorb, eliminating removal procedures and minimizing complication risks. Unlike surgical or internal medicine implants, where magnesium and zinc alloys are widely explored, wound care depends almost exclusively on organic polymer systems such as collagen, chitosan, and PLGA. These biopolymers offer precisely tunable degradation rates, exceptional biocompatibility, and the ability to locally deliver growth factors or antimicrobial agents. Their soft, hydrophilic matrices foster angiogenesis, fibroblast infiltration, and re‐epithelialization while avoiding inflammatory responses. No degradable metallic material matches their versatility or safety in delicate soft‐tissue repair, cementing organic polymer implants as the cornerstone of modern wound‐healing strategies.

#### 3.2.1 Modified Natural Polymer‐Based Biodegradable Urological Implants

Surgical wounds can precipitate substantial hemorrhage or infections, potentially culminating in diverse postoperative complications. Hence, the advancement of innovative materials that facilitate hemostasis and expedite wound healing is imperative in clinical settings. As noted repeatedly, natural and modified natural polymers, such as chitosan, cellulose, and protein, have long been a mainstay in the field of biodegradable implants. Chitosan (CS)‐based dressings have emerged as efficacious solutions for augmenting wound healing. In contrast to conventional dressings, CS dressings markedly accelerate the healing trajectory and inhibit microbial colonization. Modification of chitosan with 1‐(carboxymethyl) thymine yields thymine‐modified chitosan (TC) derivatives, exhibiting degrees of substitution ranging from 0.23 to 0.62.^[^ ^40^ ^]^ Freeze–dried, sponge‐like CS dressings demonstrated considerable efficacy in wound healing, reducing the wound area ratio from 100 % to 64.5 ± 5.2 % within three days, as opposed to a reduction to 82.5 ± 5.2 % with standard gauze dressings. Additionally, TC derivative dressings displayed broad‐spectrum antibacterial activity against both Gram‐negative and Gram‐positive bacteria, while concurrently promoting tissue regeneration and angiogenesis. The development of tough chitosan dressing (TCS), synthesized by dissolving 100 g of CS in 80 g of 75 % acetic acid employing non‐woven technology, exhibited superior antimicrobial activity and biodegradability. By optimizing the CS composition to attain a water absorption capacity of up to 2100%, TCS significantly outperformed untreated chitosan dressings (UTCS).^[^ ^103^ ^]^ Notably, TCS achieved nearly 100% antimicrobial efficacy against *Candida albicans*, surpassing both UTCS and commercially available wound dressings. Following immersion in phosphate‐buffered saline for 15 days, the dry weight degradation rate of TCS reached 43.01%, comparable to the 50% degradation rate observed for commercial alternatives. Antimicrobial efficacy is paramount for effective wound dressings. For instance, the incorporation of cinnamaldehyde has been demonstrated to augment antimicrobial activity.^[^ ^104^ ^]^ The resultant gelatin/chitosan/cinnamaldehyde crosslinked membranes exhibited potent antibacterial effects against both Gram‐positive and Gram‐negative bacteria. Incremental increases in cinnamaldehyde concentration corresponded with enhanced inhibition rates against *Pseudomonas aeruginosa* and *Staphylococcus aureus*. Furthermore, these membranes displayed excellent hemocompatibility, biodegradability, and minimal cytotoxicity, positioning them as viable candidates for biocompatible wound dressings.

**Figure 7:**
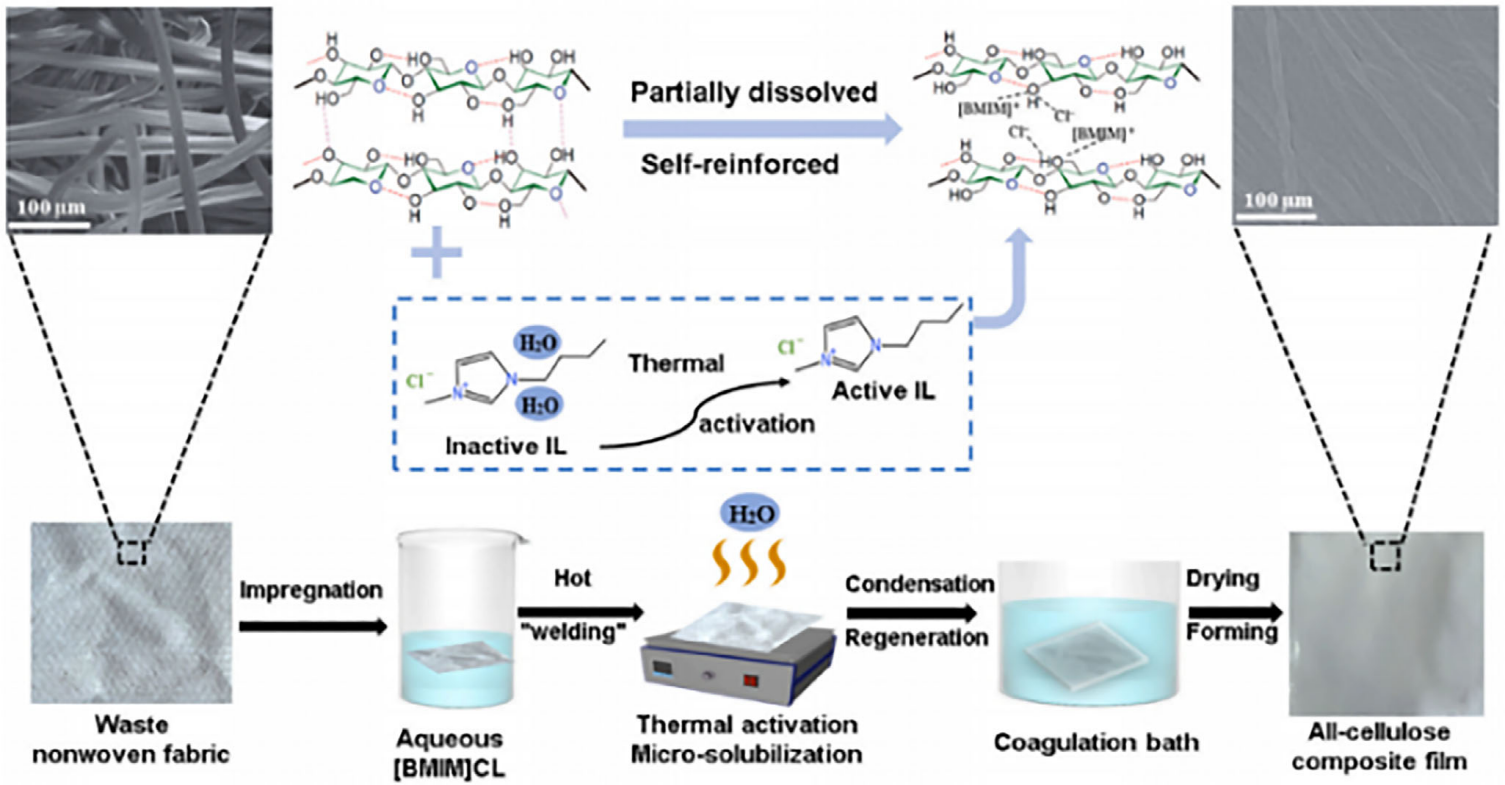
Preparation and recycling of waste non‐woven fabric. Adapte with permission.^[^ ^105^ ^]^ Copyright 2023, Elsevier Ltd.

In addition to chitosan, both cellulose‐based and protein‐based polymers are well suited for fabricating biodegradable wound‐healing implants. Researchers have engineered all‐cellulose composite films by dissolving viscose fibers in the ionic liquid 1‐butyl‐3‐methylimidazolium chloride ([BMIM]Cl) and thermally “welding” the fiber surfaces.^[^ ^105^ ^]^ These films exhibit exemplary mechanical properties (tensile strength of 25 MPa, elongation at break of 32 %), robust thermal stability, chemical resistance, 80 % light transmittance, and undergo 93.4 % degradation over a 90‐day period. This innovation offers a sustainable methodology for recycling waste cellulose into specialized materials suitable for medical applications (**Figure** **7**). In another alternative study, apricot kernel skin (AKS), abundant in antioxidants and antimicrobial compounds, was employed to fabricate degradable films with soy protein isolate to get enhanced properties.^[^ ^106^ ^]^ This investigation explored the influence of varying AKS particle sizes and loading concentrations (0 %, 10 %, 20 %, 30 % based on soy protein isolate) on the physical attributes (optical clarity, mechanical strength, water vapor permeability), as well as the antioxidant and antimicrobial activities of the degradable films. The findings revealed that films incorporating ultrafine AKS powder (UP) exhibited significantly enhanced antioxidant and antimicrobial activities. Moreover, films with a 20% UP loading achieved the highest tensile strength (7.20 MPa). This methodology presents a novel approach for developing degradable films with superior antioxidant properties. Additionally, keratinocytes have been successfully utilized to form skin equivalents. Furthermore, whey protein isolate (WPI), derived from milk, was methacrylated to produce a photo‐cross‐linkable material. This material was subsequently processed into injectable hydrogels, microspheres, and scaffolds utilizing microfluidics and 3D printing techniques.^[^ ^36^ ^]^ In vivo evaluations revealed excellent biocompatibility and biodegradability, with WPI‐MA hydrogels enhancing left ventricular function in a myocardial infarction model, thereby highlighting their therapeutic potential. This research paves the way for synergistic collaborations between the dairy industry and medical therapeutics.

#### 3.2.2 Synthetic Polymer‐Based Biodegradable Urological Implants

Polyurethanes and polyesters are two typical biodegradable materials that are broadly utilized for urological implants. In a distinct study, fully degradable soybean oil‐based waterborne polyurethane (SWPU) was synthesized, with its mechanical, thermal, and hydrophilic properties modulated by varying the R‐value.^[^ ^107^ ^]^ Soybean oil‐based polyols were reacted with isophorone diisocyanate (IPDI), dimethylolpropionic acid (DMPA), trimethylamine (TEA), and ethylenediamine (EDA) to formulate polyurethane emulsions with varying R‐values. These enhancements were attributed to improved cross‐linking within the polyurethane elastomer and stronger molecular chain interactions at elevated R‐values. The degradation behavior of SWPU films was assessed in a 3 wt% sodium hydroxide solution at 45 °C, with weight loss monitored over time. Through γ‐radiation‐induced polymerization, a brush‐like poly (2‐aminoethyl methacrylate) (PAEMA) was successfully grafted onto chitosan (CS), yielding the copolymer CS‐g‐PAEMA. This material was then processed into a sodium‐acetate‐leached poly(urethane‐urea) scaffold.^[^ ^41^ ^]^ The resulting construction demonstrated excellent biocompatibility in both in‐vitro cell‐culture assays and in Vivo animal models. Furthermore, the CS‐derived polyurethane scaffold supported highly efficient dynamic cultivation of human fibroblasts. Finally, histological and histochemical analyses in a murine study confirmed the scaffold's complete integration with the surrounding subcutaneous tissue.

The dressing industry has progressively shifted towards biodegradable alternatives, with poly (lactic acid) (PLA) emerging as a predominant polymer.^[^ ^108^ ^]^ However, the inherent high oxygen permeability of PLA films constrains their applicability. To mitigate this limitation, researchers have enhanced the oxygen barrier properties of PLA films by adjusting the thickness of both PLA and zein layers. The PLA45:15Z bilayer film exhibited significantly reduced peroxide values in sunflower oil, indicative of improved oxygen barrier performance. A recent study engineered LCE metamaterials with an innovative design, realizing a biaxial drive strain of ‐53% and a thermal expansion coefficient of −33,125 ppm K^−1^, significantly outperforming previous benchmarks.^[^ ^50^ ^]^ These materials operate effectively at lower temperatures (46 °C) while delivering high driving stresses and strains. When integrated into medical dressings, these LCE metamaterials formed a breathable, shrinkable, hemostatic patch that facilitates non‐invasive treatment. In vivo animal studies demonstrated that the patch promoted accelerated skin regeneration without scarring or hypertrophic scarring, outperforming traditional modalities such as conventional dressings and sutures.

### 3.3 Biodegradable Urological Implants

**Figure 8:**
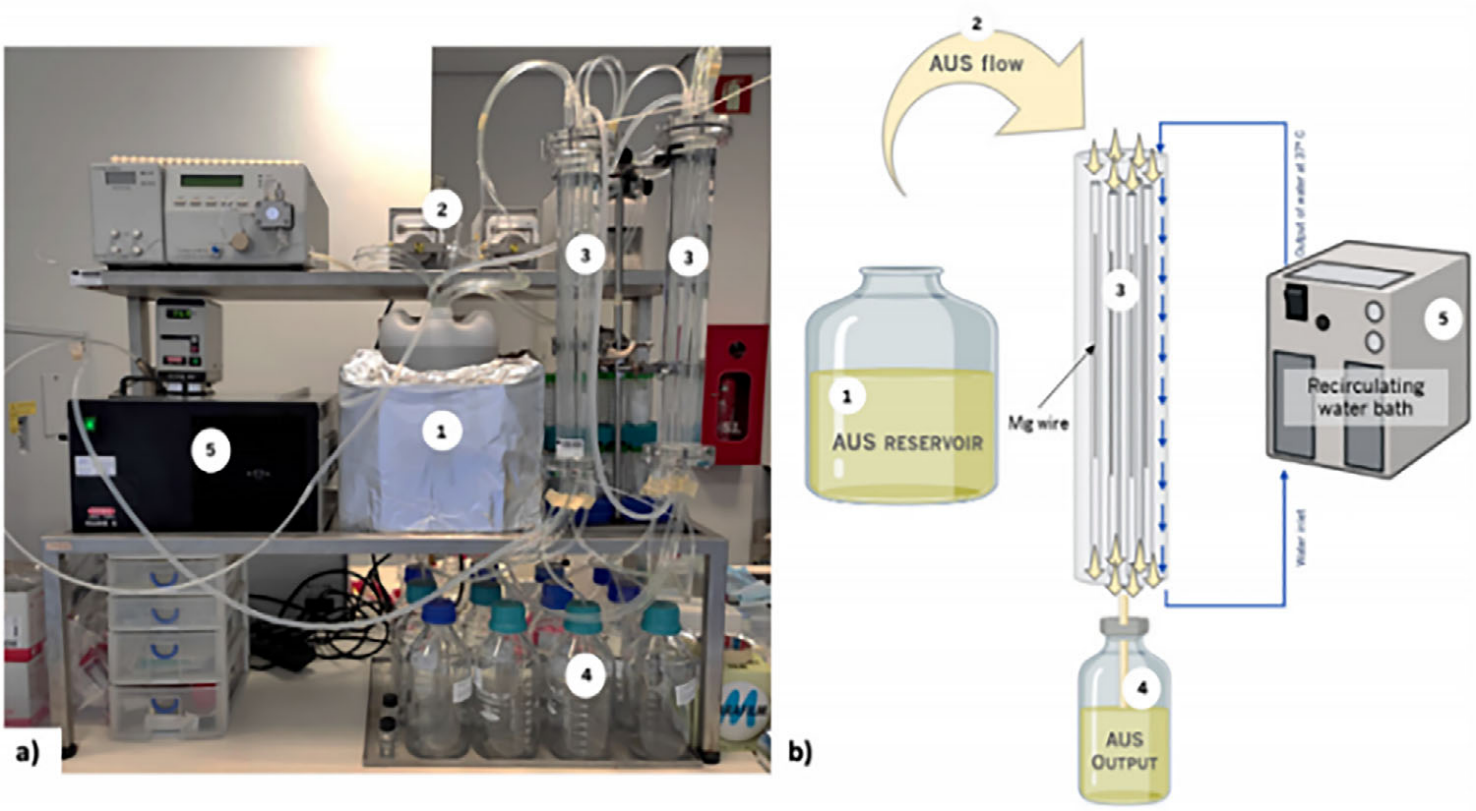
Biodegradable metals for dynamic ureteral stent system. Adapte with permission.^[^ ^109^ ^]^ Copyright 2023, Chongqing University.

Urology covers the diagnosis and treatment of urinary tract and male reproductive system disorders, including kidneys, ureters, bladder, urethra, prostate, and testes. It addresses congenital anomalies, infections, stones, tumors, incontinence, and sexual dysfunction using medical therapy, endoscopy, minimally invasive and reconstructive surgery, and transplantation. In urology, biodegradable implants have become indispensable for restoring and maintaining urinary tract function. Made from biocompatible polymers, these devices, from drug‐eluting stents to tissue‐engineering scaffolds, are precisely tailored to the urinary system's unique anatomy and physiology. By degrading in situ, they eliminate removal procedures, lower infection and encrustation risks, and enable sustained local drug delivery at obstruction or injury sites. Their ability to conform to complex ureteral and urethral geometries, support tissue regeneration, and adapt mechanically over time underscores their irreplaceable role in modern urological practice. Streamlining patient recovery and minimizing long‐term complications, biodegradable polymer implants set a new benchmark for minimally invasive, patient‐centric urological care. Ureteral stents are indispensable in contemporary urological practice; however, post‐deployment symptoms can markedly compromise patient well‐being and quality of life. In response, biodegradable metals have emerged as promising materials for stent fabrication, offering enhanced radial force and controlled degradation rates. A comprehensive study evaluated the performance of various biodegradable metals within a simulated urinary tract environment to assess their suitability for ureteral stents.^[^ ^109^ ^]^ Specifically, the corrosion behavior of five magnesium alloys‐AZ31, Mg‐1Zn, Mg‐1Y, pure Mg, and Mg‐4Ag‐was scrutinized under conditions mimicking the urinary tract. Common corrosion byproducts identified across all alloys included Mg(OH)_2_, MgO, and phosphate‐containing compounds, with slight compositional variations observed. Corrosion rates exhibited significant variability, attributable to differences in the corrosion layers, and distinct discrepancies were noted between samples subjected to stagnant versus flowing conditions. Notably, Mg‐1Zn and Mg‐4Ag demonstrated a heightened propensity for localized corrosion, likely due to the formation of less protective layers, with Mg‐4Ag exhibiting accelerated corrosion relative to other alloys. Conversely, Mg‐1Y displayed a more uniform corrosion pattern, underscoring its potential as a viable candidate for future investigations into biodegradable metals for ureteral stents (**Figure** **8**). Furthermore, the advent of degradable ureteral stents that obviate the necessity for removal procedures represents a significant advancement in urological therapeutics. Such stents could effectively mitigate the prevalent issue of “forgotten stents”, which are often associated with long‐term complications. In this context, researchers have developed a prototype biodegradable ureteral stent composed of poly(L‐lactide‐co‐ε‐caprolactone) and reported preliminary testing outcomes.^[^ ^110^ ^]^ The synthesized polymer conforms to medical standards, and mechanical assessments of the rod‐shaped stent prototypes revealed that the material exhibits both requisite strength and elasticity. Degradation assays conducted in artificial urine at 37 °C demonstrated that the stent retains its mechanical integrity for a minimum of two weeks. Additionally, cytotoxicity evaluations indicated that the material does not impede cellular proliferation, suggesting biocompatibility. These findings highlight the polymer's potential for further development and necessitate subsequent in vivo evaluations to substantiate its efficacy as a biodegradable ureteral stent.

Catheter‐associated urinary tract infections (CAUTIs) constitute approximately 40% of hospital‐acquired infections, with over one million cases reported annually. To address this significant clinical challenge, a study introduced an antibacterial hydrogel coating, AgNPs‐PAAm‐CS‐PVP, specifically designed for urinary catheters.^[^ ^111^ ^]^ The hydrogel was applied via a dipping process and incorporated silver nanoparticles averaging 25.9 nm in diameter. These nanoparticles facilitate the sustained release of antibacterial agents, thereby providing long‐term protection against *Escherichia coli*. Furthermore, the hydrogel coating effectively reduces bacterial adhesion, enhances hydrophilicity, and exhibits favorable hemocompatibility and cytocompatibility. These properties collectively position the AgNPs‐PAAm‐CS‐PVP hydrogel coating as a promising intervention for decreasing the incidence of CAUTIs. Stress urinary incontinence (SUI), defined as the involuntary loss of urine due to increased abdominal pressure during activities such as coughing or sneezing, affects approximately 20–40% of women and tends to exacerbate with advancing age. Severe cases are typically managed through the implantation of a static sling beneath the urethra; however, these slings lack the capacity for non‐invasive adjustments post‐surgery. In response, researchers have developed an innovative device fabricated from liquid crystal elastomers (LCEs) capable of altering its shape in response to infrared (IR) light.^[^ ^51^ ^]^ The device was evaluated in models simulating scar tissue and the urinary tract, demonstrating that the LCE‐based device can effectively maintain continence and modulate tension upon exposure to IR light. In vivo experiments involving rabbit models revealed that the device not only enhanced support at the bladder neck but also allowed for precise tension adjustments through IR illumination. These outcomes suggest that LCE materials hold significant promise for the dynamic treatment of SUI in women, offering adjustable support mechanisms that can be remotely controlled post‐implantation.

## 4 Biodegradable Implants for Medical Devices

### 4.1 Biodegradable Sensor

**Figure 9:**
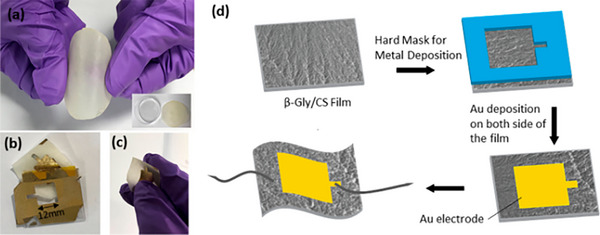
Glycine‐chitosan composite for biodegradable sensing. Adapte with permission.^[^ ^112^ ^]^ Copyright 2020 American Chemical Society.

In the medical device landscape, biodegradable sensors are increasingly recognized as essential for advanced patient monitoring. These ultra‐thin, flexible devices, fabricated from bioresorbable polymers and metals, conform intimately to tissue surfaces and provide continuous, real‐time measurements of pressure, pH, temperature, and biochemical markers. Upon completing their diagnostic function, they undergo controlled in situ degradation, obviating removal procedures, mitigating infection risk, and minimizing chronic inflammatory responses. Unlike permanent implants, biodegradable sensors reside in vivo only for the required duration, thereby streamlining clinical workflows and enhancing patient comfort. Their ability to deliver localized, high‐resolution feedback during critical healing windows before vanishing without a trace establishes them as irreplaceable tools in minimally invasive medicine. By integrating advanced sensing with inherent biocompatibility, biodegradable sensors define a new paradigm for intelligent, patient‐centric care in medical devices. A pivotal study introduced flexible, biodegradable pressure sensors constructed from glycine–chitosan piezoelectric films.^[^ ^112^ ^]^ These films are synthesized through the self‐assembly of glycine molecules within an aqueous chitosan solution, resulting in a stable β‐glycine spherulite structure embedded in the chitosan matrix. X‐ray diffraction analysis confirmed the formation of a pure ferroelectric β‐phase of glycine. Employing a straightforward solvent‐casting methodology, the researchers produced biodegradable piezoelectric films with a sensitivity of approximately 2.82 ± 0.2 mV kPa^−1^, comparable to that of non‐degradable commercial counterparts. The films exhibited capacitance values ranging from 0.26 to 0.12 nF and demonstrated dielectric properties favorable for integration into wearable biomedical sensors. These findings underscore the expanding potential of biodegradable piezoelectric materials in energy harvesting, biosensing, and implantable devices, thereby establishing a robust foundation for the progression of green electronics and precision medicine (**Figure** **9**). Poly (L‐lactic acid) (PLLA), particularly in its electrospun nanofibrous form, has garnered considerable attention due to its inherent biodegradability.^[^ ^113^ ^]^ Nonetheless, the mechanisms underpinning their piezoelectric properties remain only partially elucidated. Utilizing a design‐of‐experiment approach, researchers investigated the impact of fiber diameter and thermal treatment on the piezoelectric performance of electrospun PLLA nanofibers. The results revealed that alterations in phase content, transitioning from amorphous to crystalline α/α’ phases, differentially influence piezoelectric behavior. Furthermore, the piezoelectric properties of PLLA nanofibers were observed to modulate stem cell differentiation, thereby promoting neurogenesis and osteogenesis. These outcomes suggest that PLLA nanofibers with meticulously controlled piezoelectric properties could serve as effective self‐powered platforms for stem cell engineering.

Further exploration into biodegradable energy harvesters has been undertaken using electrospun silk nanofiber membranes.^[^ ^114^ ^]^ The transition of silk fibroin from an α‐helix to a β‐sheet conformation, induced by ethanol treatment, alters the molecular dipole moment and subsequently affects piezoelectric properties. Notably, the conventional poling process, typically employed to enhance piezoelectricity, was found to attenuate the piezoelectric response of silk fibroin. This diminution is attributed to the disruption of hydrogen bonds under high electric fields, leading to phenomena termed “inverse depolarization” and “quasi‐piezoelectricity” in protein‐based biopolymers. The study successfully demonstrated silk‐based piezoelectric generators, thereby confirming their biodegradability and efficacy in self‐powered motion‐detecting sensors. These findings provide critical insights into the piezoelectric behavior of silk fibroin and present high‐performance energy harvesting devices tailored for specific sensor applications.

### 4.2 Other Biodegradable Medical Devices

**Figure 10:**
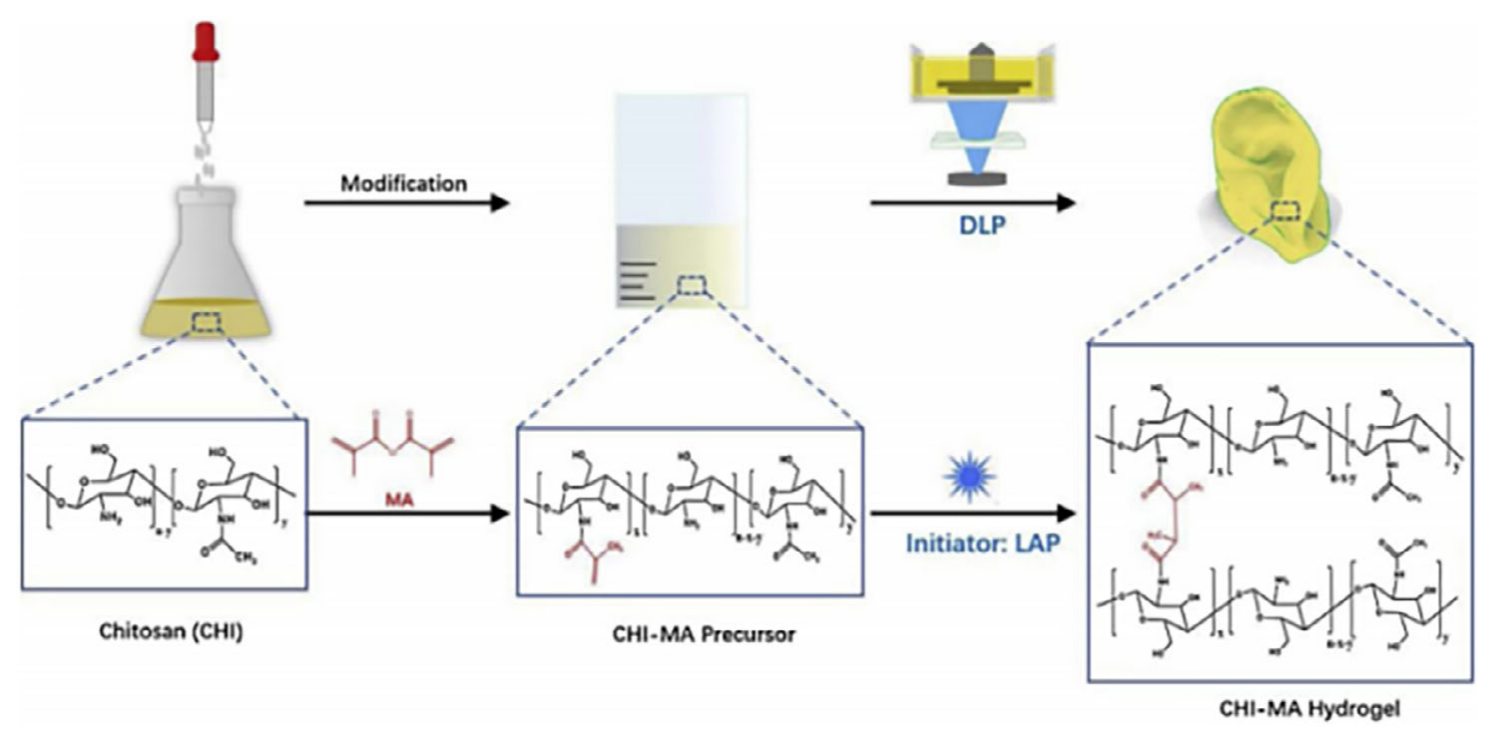
Schematic representation shows the chemical functionalization of chitosan with methacrylate groups and the photocuring process of CHI‐MA hydrogels. Adapte with permission.^[^ ^116^ ^]^ Copyright 2020, Elsevier Ltd.

The continued development of biodegradable medical devices, which can be effectively monitored using rapid and non‐invasive imaging modalities, is crucial for the successful treatment of various pathological conditions. Recent advancements have led to the creation of a 3D‐printable radiopaque polymer specifically designed to facilitate the tracking of biodegradable implants via imaging techniques such as X‐ray and computed tomography (CT).^[^ ^115^ ^]^ This polymer, poly (glycerol sebacate) acrylate (PGSA), was chemically modified with barium sulfate, bismuth subcarbonate, and bismuth oxychloride to enhance its radiopacity. Among these modifications, PGSA‐BiOCl was selected for in vitro degradation assessments over a thirty‐day period, revealing a direct correlation between mass loss and diminished radiopacity. These findings highlight the polymer's potential utility in real‐time monitoring of implant degradation through imaging technologies. Concurrently, researchers have developed a photocurable chitosan bioink (CHI‐MA) optimized for digital light processing (DLP) technology.^[^ ^116^ ^]^ The synthesis of CHI‐MA involved grafting methacryloyl groups onto chitosan molecular chains, thereby enabling photo‐crosslinking. The study meticulously evaluated the influence of variables such as concentration and degree of substitution (DS) of CHI‐MA on the rheological properties, photocuring kinetics, and mechanical characteristics of the resultant photo‐crosslinked gels. A high degree of substitution (33.6%) effectively minimized the curing time required to fabricate a 150 µm thick hydrogel layer. Cytotoxicity assays confirmed that both the photocuring process and the resulting hydrogels exhibited exceptional biocompatibility. Utilizing DLP printing, the CHI‐MA bioink was successfully fabricated into intricate 3D hydrogel structures, achieving high resolution, fidelity, and biocompatibility, thereby positioning it as a promising candidate for advanced DLP‐based bioprinting applications (**Figure** **10**).

Furthermore, injectable conductive hydrogels (ICHs) have been engineered as implantable bioelectrodes with tunable degradability.^[^ ^52^ ^]^ These hydrogels, synthesized via thiol‐ene chemistry, exhibit commendable conductivity (21–22 mS cm^−1^), biocompatibility, and adjustable degradation profiles. Specifically, hydrolyzable variants degrade within three days, while more stable formulations maintain integrity for up to seven days. In vivo evaluations conducted on rodent models demonstrated that ICHs provided superior electromyography signal sensitivity compared to conventional skin electrodes and nonconductive hydrogel counterparts. These attributes render ICHs highly promising for bioelectronic applications, owing to their controllable degradation rates and efficient signal transmission capabilities. Additionally, a bio‐based eugenol epoxy was grafted onto polymethylhydrosiloxane (PMHS‐x) to reduce its dielectric constant (D_k_) and impart degradability.^[^ ^117^ ^]^ The curing process of these bio‐based silicone/epoxy resins was thoroughly investigated, revealing low Dk values and enhanced hydrophobicity. The resin exhibited degradability under alkaline conditions, thereby facilitating fiber recycling processes. Quartz fiber‐reinforced composites fabricated with this resin demonstrated a 26.5% increase in impact strength and a 16.7% reduction in Dk compared to conventional epoxy composites. These hybrid resins present an environmentally sustainable solution for applications in microelectronic devices and fiber recovery, underscoring their significance in advancing sustainable material science.

## 5 Potential Biodegradable Implant Materials

In the domain of biodegradable implants, several advanced materials remain at a preclinical stage despite demonstrating exemplary biocompatibility, predictable degradation kinetics, and unique functionalities, such as stimuli‐responsiveness and intrinsic bioactivity. Biodegradable elastomers, resins, and polyesters exhibit favorable mechanical properties, tunable resorption profiles, and capacities for controlled drug release and in vivo sensing. These nascent materials offer considerable promise as platforms for next‐generation implants, enabling dynamic tissue integration, on‐demand therapeutic delivery, and continuous physiological monitoring. Targeted research and development efforts aimed at optimizing their composition, processing methods, and regulatory compliance can translate these candidates into safe, high‐performance biodegradable devices. Such progress will drive transformative advances in regenerative medicine and minimally invasive therapies, highlighting the critical role of novel biodegradable materials in the future of medical implants.

### 5.1 Biodegradable Elastomer

Degradable biomedical elastomers (DBEEs) represent a forefront in bioremediation materials, owing to their controlled biodegradability, biocompatibility, customizable elasticity, and superior processing capabilities. Recent investigations have delved into the degradation mechanisms, synthesis and crosslinking methodologies, microstructural optimization, and advanced processing techniques‐such as solvent casting, electrospinning, and 3D printing‐of DBEEs. These studies underscore the significant potential of DBEEs in regenerating and repairing diverse tissues, including neural, tendinous, muscular, cardiac, and osseous structures.^[^ ^118^ ^]^ Beyond DBEEs, elastomeric biopolymers like poly (glycerol sebacate) (PGS) have been extensively explored for tissue engineering scaffolds. PGS, synthesized from sebacic acid and glycerol, exhibits promising attributes; however, it faces limitations regarding its inherent properties.^[^ ^119^ ^]^ To address these limitations, recent research has incorporated gelatin‐a biocompatible and bioactive polymer‐into the PGS matrix, thereby enhancing its physicochemical characteristics. These PGS‐based composites were further reinforced through in‐situ polymerization with gelatin and the addition of graphene oxide and clay nanoparticles. Comprehensive characterization revealed notable improvements in structural integrity, morphological uniformity, hydrophilicity, thermal stability, degradation kinetics, and swelling behavior. The synergistic interactions among gelatin, graphene oxide, and clay nanoparticles effectively fine‐tune the material properties and strengthen the microstructural framework, thereby broadening the functional applications of PGS‐based elastomers. Achieving muscle‐like actuation in liquid crystal elastomers (LCEs) necessitates precise mesogen alignment, typically fixed chemically during synthesis.^[^ ^120^ ^]^ A novel dual‐phase LCE network incorporating a crystalline phase that melts above the liquid crystalline transition temperature has been introduced. This crystalline phase serves as an “alignment frame”, stabilizing mechanical deformations through a shape memory mechanism that aligns mesogens within the liquid crystalline phase. Importantly, this alignment can be erased by melting, allowing for rapid reprogramming within seconds. This innovative approach offers greater flexibility compared to traditional methods and facilitates the design of 3D‐printed LCEs with multiple programmable actuation modes.

**Figure 11:**
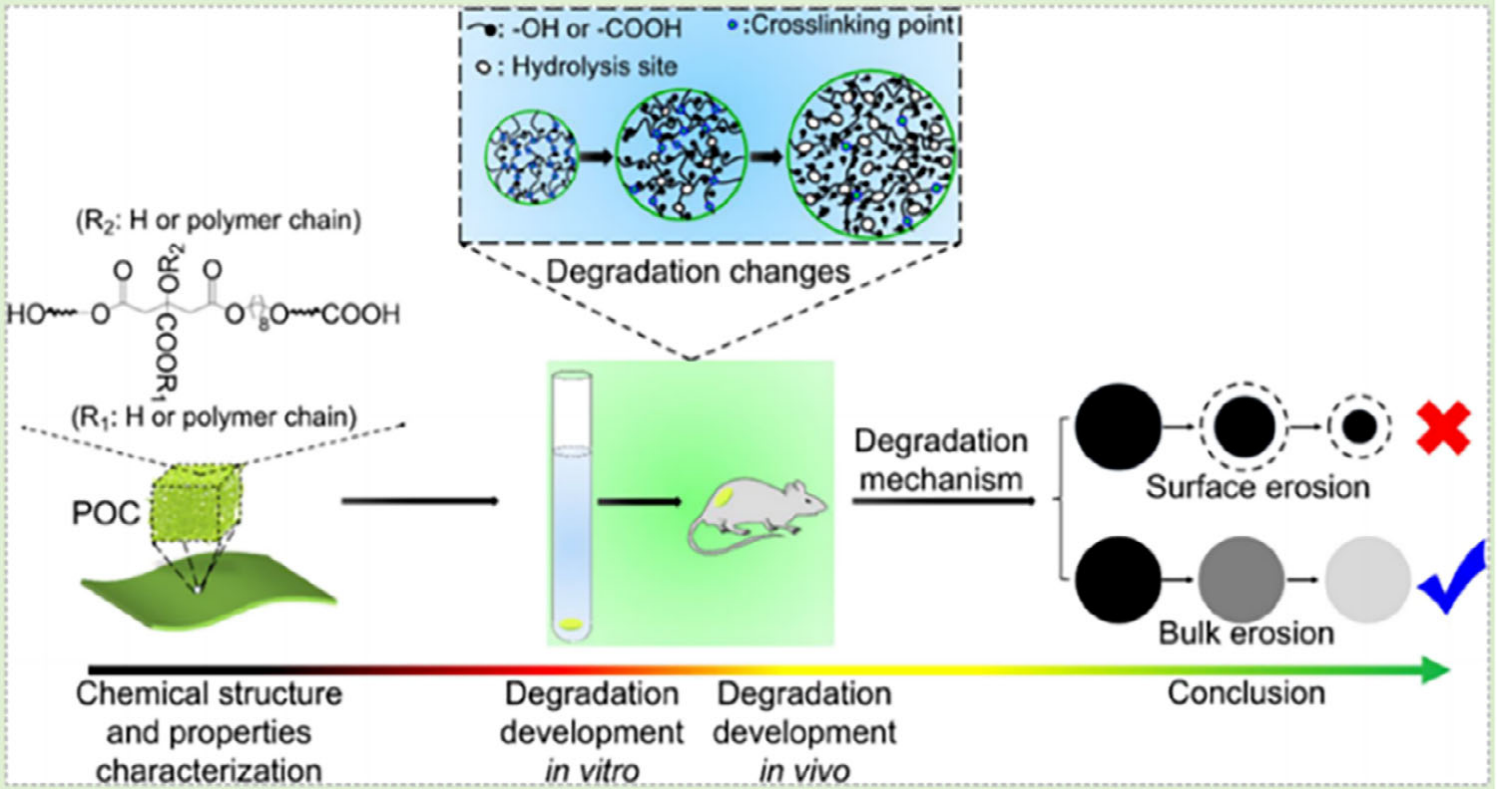
Degradation predominantly occurs through bulk erosion and is associated with a mild inflammatory response. Adapte with permission.^[^ ^121^ ^]^ Copyright, 2022 American Chemical Society.

Poly(1,8‐octanediol‐co‐citrate) (POC) is a biodegradable polymer extensively utilized in tissue engineering and implantable electronics. However, its degradation mechanism has not been thoroughly elucidated. A comprehensive study examined POC's degradation under both laboratory and in vivo conditions, revealing a gradual loss of cross‐linking density, water absorption, surface roughening, swelling, and fragmentation, culminating in an early reduction in mechanical properties. The degradation predominantly occurs through bulk erosion and is associated with a mild inflammatory response, providing critical insights for optimizing POC's biomedical applications (**Figure** **11**).^[^ ^121^ ^]^ Additionally, silver‐doped mesoporous bioactive glass/poly (1,8‐octanediol citrate) (AgMBG/POC) elastomeric biocomposite scaffolds were fabricated using salt‐leaching techniques for the first time.^[^ ^122^ ^]^ Varying concentrations of AgMBG (5, 10, and 20 wt.%) were evaluated, demonstrating a uniform distribution within the POC matrix. At 20 wt.%, AgMBG significantly enhanced thermal stability, mechanical properties, and water uptake. Moreover, the 20 wt.% AgMBG/POC scaffold exhibited an accelerated degradation rate, losing approximately 26% of its initial weight after 28 days in Tris‐HCl. These scaffolds also demonstrated effective antibacterial activity against *Escherichia coli* and *Staphylococcus aureus*, while maintaining good biocompatibility with human dermal fibroblasts. The integration of AgMBG with POC highlights the potential of these scaffolds for soft tissue engineering applications.

### 5.2 Biodegradable Resin

**Figure 12:**
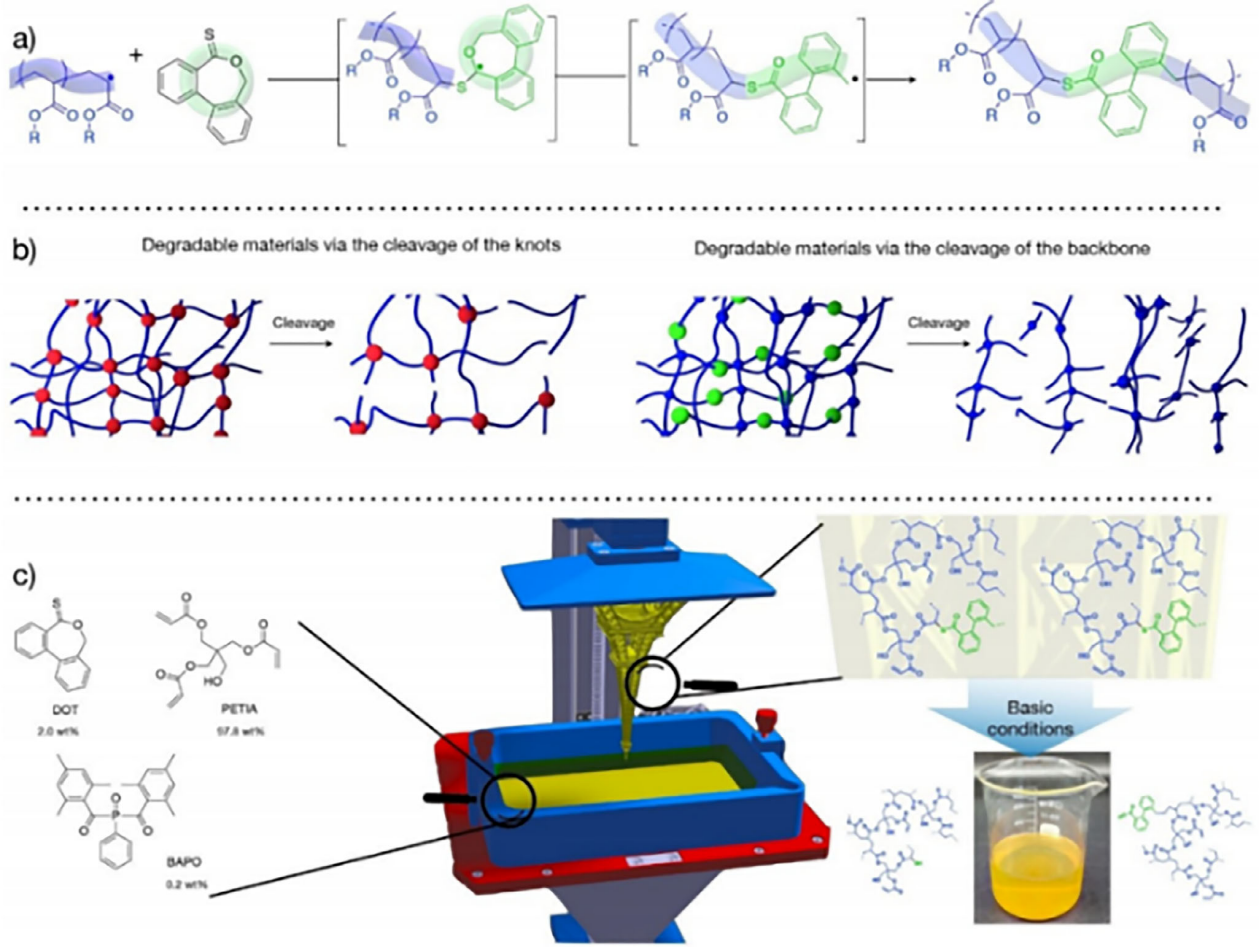
Schematic representation of the writing/erasing process. Adapte with permission.^[^ ^124^ ^]^ Copyright, 2022 The Authors.

Gelatin methacryloyl (GelMA), a modified extracellular matrix (ECM)‐derived biopolymer, is widely utilized in the fabrication of 3D tissue engineering scaffolds. However, GelMA's propensity for physical gelation restricts its application in aqueous 3D printing resins, often resulting in reduced resolution during stereolithography (SLA). To mitigate this limitation, researchers developed GelMA‐based resins derived from fish and porcine sources using formamide as a solvent, either with GelMA alone or in combination with star‐shaped poly(ε‐caprolactone) methacrylate (PCL‐MA).^[^ ^123^ ^]^ Optimal formulations, including GelMA resins and GelMA/PCL‐MA hybrid resins at a 70/30 wt.% ratio, were identified to achieve appropriate viscosity for SLA at 32 °C. These formulations were successfully employed to 3D print tissue scaffolds replicating the human small intestine. Beyond GelMA‐based systems, enhancing the degradability and recyclability of 3D‐printed materials remains a significant challenge. Traditional VAT photopolymerization produces cross‐linked materials with high thermal, chemical, and mechanical stability, which are not ideal for degradable applications. To address this, a study introduced 2 % thionolactone, specifically dibenzo[c,e]‐oxepane‐5‐thione (DOT), into acrylate‐based resins. This additive facilitates weak bond formation through radical ring‐opening polymerization, thereby enhancing the resin's degradability without significantly affecting printability, resolution, or mechanical properties.^[^ ^124^ ^]^ Resins containing DOT were employed in UV microfabrication, two‐photon stereolithography, and commercial 3D printing systems. The resultant 3D objects demonstrated degradation in basic solvents and homemade compost, with the degradation rate being dependent on the object size. This property was leveraged to fabricate 3D structures with support components that could be easily solubilized (**Figure** **12**). Collectively, advancements in GelMA/PCL‐MA hybrid resins and the incorporation of DOT into acrylate‐based systems represent significant progress in developing biodegradable resins for 3D printing. These innovations enhance printing fidelity, biocompatibility, and degradability, thereby expanding the application scope of 3D‐printed scaffolds in tissue engineering and other biomedical fields.

### 5.3 Biodegradable Polyester

Polyesters are highly regarded as biodegradable materials due to their environmentally sustainable properties, which facilitate degradation over time and thereby reduce long‐term ecological impact. Their exceptional versatility, mechanical robustness, and suitability for a wide array of applications make them particularly valuable in the realms of medical devices, tissue engineering, and sustainable packaging. In tissue engineering, researchers have developed rapidly photocurable polyurethanes (PUs) by utilizing poly(ε‐caprolactone) (PCL) and polyethylene glycol (PEG) as microdiols to fabricate elastic 3D scaffolds.^[^ ^125^ ^]^ Evaluation using MTT and AlamarBlue assays on dermal fibroblast cells revealed significant cellular proliferation on the printed PU/PCL/PEG scaffolds, with no indications of cytotoxicity. Furthermore, compared to cast film PUs, the printed PU/PCL/PEG scaffolds demonstrated enhanced cell adhesion on their surfaces even after four days of incubation, highlighting their high potential for soft tissue engineering applications, particularly in scaffold development.

In a complementary study, α‐(Chloromethyl) acryloyl chloride was polymerized with various bisphenols and diamines to synthesize poly (conjugated esters).^[^ ^126^ ^]^ These poly conjugated esters were subsequently subjected to chemical degradation via main‐chain scission through conjugate substitution with benzyl mercaptan. Notably, the chemical degradation and bisphenol recovery processes can be efficiently executed in aqueous environments under ambient conditions, enhancing the material's applicability for both positive‐ and negative‐resist applications, as well as partially renewable polymer systems. Although 3D printed hydrogel scaffolds have demonstrated considerable promise across various applications, their utilization in regenerating damaged or deficient adipose tissue (AT) remains relatively underexplored. Due to their structural integrity, biodegradability, and mechanical properties analogous to soft tissues, 3D‐printed hydrogels are ideally suited for the aesthetic and functional restoration of AT. In this context, a straightforward and cost‐effective 3D printing methodology was developed utilizing gelatin‐based ink to fabricate scaffolds for AT engineering.^[^ ^127^ ^]^ The ink, composed of gelatin and methylenebisacrylamide as a crosslinking agent, was extruded via pneumatic 3D printing. Subsequent crosslinking at varying temperatures enhanced scaffold stability, with those crosslinked at 20 °C maintaining structural integrity for 21 days under physiological conditions. These scaffolds exhibited the adhesion and proliferation of human pre‐adipocytes and supported their differentiation into adipogenic lineages, thereby validating the potential of gelatin‐based hydrogels as scaffolds for AT regeneration. In a separate investigation, the integration of two‐photon polymerization (2PP) into tissue engineering and regenerative medicine (TERM) has garnered significant interest due to its capacity to fabricate highly intricate scaffolds with customizable architectures. Researchers introduced the first biodegradable and biocompatible poly (trimethylene carbonate) (PTMC)‐based scaffold fabricated using 2PP, achieving substantial dimensions of 18 × 18 × 0.9 mm and a volumetric capacity of 292 mm^3^.^[^ ^128^ ^]^ This represents a remarkable enhancement in volumetric scalability compared to previously available large‐scale structures. The scaffold exhibited a porosity of 96% and demonstrated superior cytocompatibility, promoting the attachment, proliferation, and differentiation of human adipose‐derived mesenchymal stem cells into osteogenic and chondrogenic lineages.

### 5.4 Other Biodegradable Implants Materials

The synthesis of robust gelatin hydrogels has been advanced through pullulan dialdehyde (PDA) as a macromolecular crosslinker.^[^ ^129^ ^]^ Gelatin hydrogels crosslinked with PDA (G‐PDA) achieved compressive stress of 5.80 MPa at 80% strain, a 152‐fold increase over unmodified gelatin. FTIR and SEM confirmed PDA incorporation, and a ninhydrin assay quantified crosslinking, revealing that dual‐crosslinking (covalent and reversible interactions) elevates load‐bearing capacity. Increasing PDA reduced swelling and enzymatic degradation, enhancing stability. MTT assays showed G‐PDA hydrogels are non‐cytotoxic to MC3T3 cells at all PDA levels. Chitosan, known for biocompatibility, biodegradability, and support of cell growth, was used to fabricate chitosan‐alginate polyelectrolyte complexes (PECs) by freeze‐drying and CaCl_2_ crosslinking.^[^ ^130^ ^]^ The CS1‐AL2.3 scaffold exhibited 8 MPa strength and 100–150 µm pores. Further crosslinking raised density and elastic modulus to 30 MPa. Post‐treatment with hyaluronic acid–conjugated fibrinogen and functionalization with pyridine‐2‐sulfonate or heparin improved antioxidant properties, porosity, hydrophilicity, and fibroblast adhesion, highlighting their promise for tissue engineering.

## 6 Limitations and Future Perspectives

Although biodegradable medical materials hold great promise for advancing patient care, their widespread clinical translation over the past five years has been hampered by several persistent challenges. These include inconsistent degradation rates that fail to match tissue‐healing timelines; insufficient mechanical strength for load‐bearing applications; potential inflammatory or toxic responses to degradation by‐products; limited alloy performance or poor solubility in certain polymers; complex and costly manufacturing processes; and a lack of standardized degradation protocols and long‐term clinical data. Moreover, current implants rarely incorporate dynamic functionalities, such as real‐time adaptation to pH shifts or mechanical stresses, needed to meet the demands of living tissues. A focused review of recent developments highlights three critical limitations:

a) Application and material imbalances

As shown in this review, surgical applications predominate (56% of studies), far outstripping internal implants (17%) and medical devices (7%); furthermore, orthopedic use constitutes 75% of the surgical subset. A parallel skew is evident in material choice: degradable metal alloys account for over half of reported orthopedic implants, while investigations of natural and modified natural polymers remain scarce. Synthetic‐polymer research is likewise concentrated on conventional polyesters (PLA, PCL, PLGA), leaving many novel degradable polymers unexplored for implantable use. Rectifying these imbalances through diversification of both application scenarios and material systems will be critical to fully realize the clinical potential of biodegradable implants.

b) Insufficient in vivo validation

Despite considerable advances in material design, most studies of biodegradable implants remain confined to in‐vitro evaluation, with only 44% incorporating in vivo analyses. This paucity of animal experiments markedly limits the comprehensive assessment of long‐term biocompatibility, degradation kinetics under physiological conditions, and functional performance within complex tissue environments. In the absence of robust in vivo validation, critical insights into host‐implant interactions, immunological responses, and mechanical stability over time are lacking, thereby impeding regulatory approval and clinical translation. Systematic preclinical evaluation in appropriate animal models is therefore essential to refine implant formulations and accelerate their safe adoption in patient care.

c) Poorly understood and uncontrolled degradation behavior

In metallic alloys, corrosion typically proceeds via coupled anodic and cathodic reactions, whereas hydrolysis dominates the breakdown of organic polymers, such as polyesters, polylactic acids, proteins, and polysaccharides. However, the fate and biological impact of the resulting degradation products demand urgent, systematic study. Adding further complexity, implants are placed in anatomically distinct sites, each with unique pH, enzymatic activity, and mechanical loading, so strategies to fine‐tune degradation rates in situ are critically needed. Interestingly, recent advances in microplastics research have illuminated how polymer fragments interact with human tissues and provoke inflammatory or cellular responses. By adapting analytical techniques and mechanistic insights from the microplastics field, researchers can more accurately track implant fragment formation, assess biocompatibility of by‐products, and ultimately design biodegradable devices with predictable, site‐specific degradation profiles for safer clinical translation.

In general, biodegradable implants are driving a seismic shift in internal‐medicine therapies by doing away with the very drawbacks that have long haunted permanent metal devices. Instead of leaving behind a lifetime of foreign material, these ingenious constructions offer temporary mechanical support that gradually degrades as tissue healing progresses. By eliminating the need for secondary removal surgery, these implants reduce infection risk, patient discomfort, and overall healthcare costs, which is reshaping future medical practice. Facing this key factor, future work should be concentrated on three aspects:

- Mechanistic clarity. Precisely defining degradation pathways and mapping the biological effects of every by‐product will enable truly predictive, site‐specific resorption profiles.
- Cost‐performance balance. Affordability is as crucial as biocompatibility. Developing high‐quality implants at sustainable prices will ensure broad patient access and real cost savings for healthcare systems.
- “Materials as medicines”. Embedding bioactive units, antibacterial, anti‐inflammatory, or even pro‐regenerative moieties, into the implant matrix transforms passive scaffolds into therapeutic platforms, achieving synergistic benefits greater than the sum of their parts.

Moreover, emerging nanofabrication technologies will be pivotal for the next generation of biodegradable implants. Techniques such as nanoscale surface patterning, nanoporous network fabrication, and bottom‐up self‐assembly provide unprecedented control over microstructure, degradation kinetics, and local drug or ion release. By tailoring feature sizes to the 10‐100 nm range, we can finely tune protein adsorption, cell adhesion, and immune signaling at the implant‐tissue interface. When integrated with our three interwoven strategies, total mechanistic insight, economic viability, and built‐in bio‐functionality, these nanofabrication approaches will transform biodegradable implants from clever engineering feats into indispensable tools of tomorrow's medicine.

## 7 Conclusion

Biodegradable medical materials are revolutionizing biomedical engineering by providing versatile solutions for tissue regeneration, implantable devices, and sustainable medical applications. This review has examined a range of materials, including degradable elastomers, GelMA‐based resins, biodegradable polyesters, and specialized hydrogels, highlighting their advancements in mechanical properties, biocompatibility, and functional adaptability. These materials have demonstrated effective regeneration of tissues such as neural, cardiac, and bone, underscoring their pivotal role in regenerative medicine. However, several challenges impede their widespread clinical adoption. Inconsistent degradation rates, inadequate mechanical strength for load‐bearing applications, and potential inflammatory responses from degradation byproducts remain significant obstacles. Additionally, manufacturing complexities, high costs, and the absence of standardized degradation protocols contribute to regulatory uncertainties. Addressing these issues requires enhancing material properties through strategies like blending polylactic acid with natural fibers and employing machine learning for predictive degradation modeling. This review synthesizes current advancements and identifies critical gaps, serving as a valuable resource for researchers and practitioners. By consolidating diverse studies, it provides actionable insights and future research directions to drive innovation and facilitate the translation of biodegradable materials from the laboratory to clinical settings. Looking forward, interdisciplinary collaboration and the development of multifunctional platforms that integrate structural support, bioactive signaling, and controlled therapeutic delivery are essential. Overcoming existing challenges will enhance clinical outcomes and promote sustainable medical practices, paving the way for next‐generation regenerative therapies. In summary, biodegradable medical materials hold immense potential to advance personalized and eco‐friendly medical solutions, making this review a crucial contribution to the field.

## Conflict of Interest

The authors declare no conflict of interest.

## Author Contributions

B.X. and Y.L. contribute equally to this work. Z.W.S, Y.L., and Y.K. Xing contributed to the original draft and carried out the investigation. X.C. Pan and Z.W. Shi were involved in the investigation and secured funding. All authors have read and agreed to the published version of the manuscript.
